# A Diffusion-Based Digital Twin for Predicting Road Deterioration under Climate and Traffic Stress

**DOI:** 10.34133/research.1417

**Published:** 2026-09-23

**Authors:** Ning Pan, Yuchuan Du, Ajith Kumar Parlikad

**Affiliations:** ^1^ Department of Engineering, University of Cambridge, Cambridge, UK.; ^2^ Key Laboratory of Road and Traffic Engineering of the Ministry of Education, Tongji University, Shanghai, China.

## Abstract

Accurately tracking the spatiotemporal evolution of road distress is essential for formulating informed repair and maintenance strategies. However, existing road performance prediction approaches typically rely on weighted composite indices such as the Pavement Condition Index (PCI) rather than fine-grained, distress-level observations. This coarse representation limits the ability to detect early-stage deterioration and constrains the development of precise, predictive maintenance models. To address these limitations, this study proposes a physics-embedded diffusion (PED) model that provides a physically consistent spatiotemporal framework for jointly modeling distress evolution, maintenance interventions, and environment-induced disturbances. Validation using real-world datasets from China demonstrates strong agreement between PED predictions and observations, achieving a mean PCI mean absolute percentage error (MAPE) of 9.262% and a mean weighted percentage error of area (area MWPE) of 4.484%. Furthermore, evaluation on the US Long-Term Pavement Performance dataset confirms the model’s strong cross-regional generalization capability, achieving a mean PCI MAPE of 28.054% and an area MWPE of 8.957%, underscoring the robustness and transferability of the proposed framework. Experimental results further indicate that the optimal historical environmental window for short-term distress prediction is approximately 50 d. As the volume of training data increases, the model continues to maintain stable convergence performance, demonstrating the scalability and robustness of the proposed physics-guided deterioration modeling approach. This scalable tool is invaluable for simulating realistic road deterioration processes, providing a reference for evaluating subsequent maintenance decision-making strategies.

## Introduction

The performance of road infrastructure, shaping both its safety and accessibility, directly governs the quality of experience for every road user [1]. The deterioration of road infrastructure poses a marked challenge for transportation agencies worldwide. According to the ASCE 2025 Infrastructure Report Card, in the United States, 39% of major roads are rated in poor or mediocre condition, and deteriorated and congested roads cost the average American driver over US$1,400 annually in extra vehicle expenses and lost time due to delays [2]. Similarly, in the United Kingdom, recent RAC Foundation reports show that a substantial share of the network remains in substandard condition, with 12% of roads in England (excluding London), 19% in London, and 8% in Wales requiring maintenance within the next year. Despite regular funding, the estimated one-time “catch-up” cost has risen to £16.8 billion in 2025, and clearing the maintenance backlog is projected to require 10 to 13 years across regions. In the 2024 to 2025 period, local authorities repaired 1.9 million potholes at a cost of £137.4 million, equivalent to one repair every 17 s, underscoring the considerable operational pressure and the predominantly reactive nature of current maintenance practices [3].

These figures also highlight the urgency of improving road monitoring and predictive maintenance strategies, particularly in the context of rising urban traffic volumes and increasingly unpredictable climate conditions [4]. Road deterioration is a complex process influenced by both intrinsic and extrinsic factors, including environmental conditions [5], traffic loads [6], and material properties [7]. The interdependency of these factors over both short-term and long-term [8] periods has posed challenges to the development of robust predictive models. Climate change may further exacerbate these challenges by accelerating road degradation. Rising temperatures, increased frequency of extreme weather events, and shifting precipitation patterns [9] have been shown to intensify distress mechanisms [10]. For instance, elevated temperatures promote rutting and cracking, while extreme events such as flooding or droughts alter moisture dynamics and increase freeze–thaw cycles, which may worsen overall road conditions [11]. Recent studies have highlighted the importance of integrating climate change scenarios into predictive frameworks to assess future road conditions under varying environmental stressors [12]. Such models can identify vulnerable road sections and support the formulation of climate-resilient maintenance strategies. In addition to environmental stressors, vehicle load characteristics also substantially affect road performance. Factors such as vehicle weight, traffic density, and axle configurations impose cumulative stresses on road structures. As vehicle fleets evolve, it becomes increasingly important to understand how such transitions will influence road deterioration [13]. This necessitates the development of dynamic predictive models that can accommodate evolving load patterns. Incorporating traffic load data into these models enables more accurate estimation of road lifespan [14].

Distinct yet inherently interrelated mechanisms govern the evolution of road distress. Specifically, transverse cracking is primarily climate driven, typically associated with temperature-induced shrinkage [15], whereas fatigue-related distresses such as alligator cracking are predominantly load driven due to repeated traffic loading [16]. Longitudinal cracking is often linked to structural weaknesses and traffic-induced stresses. Pothole formation, in contrast, generally arises from coupled effects, where environmental factors (e.g., moisture) interact with traffic loading to accelerate material disintegration. In addition to the independent occurrence of individual-distress types, certain forms of distress may evolve through transitions driven by environmental factors across different spatiotemporal scales. This process can be conceptualized as a multistage causal pathway under natural deterioration conditions. At the microscale and early stage, environmental stresses induce material degradation and the initiation of microcracks. At the transitional scale, the accumulation of microcracks under sustained loading leads to their interaction and propagation into alligator cracking over longer time periods. At the macroscale, these progressively deteriorated regions become more vulnerable to external disturbances, where short-term extreme events (e.g., sudden loading or adverse weather) can act as triggering factors, leading to localized failures such as potholes [17]. These processes manifest across multiple spatial levels. At the distress level, distress evolves through transitions between different forms (e.g., from microcracks to alligator cracking and potholes). At the segment or region level, the spatial distribution and interaction of multiple distress instances influence localized road performance. At the network level, the aggregated effects of such deterioration patterns determine overall system condition and maintenance priorities, which will in return influence future performance [18].

Despite considerable investments in road management systems, most conventional field inspection approaches still rely on coarse-grained data collection, typically on an annual or semiannual basis [19], making it difficult to detect early-stage distress or capture the short-term dynamics of road deterioration. This low-frequency observation cycle severely limits the capacity for proactive maintenance planning and hinders the development of models that reflect the evolution of individual distress. In addition, most field inspection data provide valuable real-world insights but are typically aggregated into composite indices like the Pavement Condition Index (PCI). These indices rely on subjective weighting of various distress types, limiting precision and interpretability [20]. While these indices provide a coarse summary of road health, they obscure the localized behavior and microscale evolution of individual-distress types [21]. Therefore, they fail to capture how distress initiates, expands, or interacts across different spatial regions, particularly under short-term external influences like traffic or maintenance interventions.

On the other hand, traditional laboratory evaluations of road distress evolution are typically microscopic, involving prefabricated road specimens subjected to controlled environmental conditions to simulate the development of a specific type of distress [22]. While these experiments offer valuable information, they simplify environmental conditions and fail to represent real-world complexity. In addition, road performance models have employed deterministic statistical methods, such as negative exponential distributions [23], to describe deterioration trends. While useful for long-term planning, these models often fail to capture the stochastic variability inherent in real-world scenarios, especially under continuous maintenance and changing environmental conditions [24].

Emerging technologies, such as machine learning (ML) and advanced image processing, offer promising alternatives by enabling fine-grained and objective analysis of high-resolution monitoring data from diverse sensor sources [25]. Recent advances in automated road defect detection, particularly those based on deep learning architectures such as YOLO variants, have enabled real-time and high-resolution identification of road distresses, providing richer and more fine-grained data sources for predictive modeling frameworks [26]. Attention mechanisms can be leveraged to further enhance the accuracy of fine-grained distress observation [27]. In addition, comprehensive surveys on automated crack detection methods further highlight the growing capability of extracting detailed distress information from heterogeneous monitoring systems [28]. Deep learning [29] has shown potential in extracting meaningful features from complex visual or spatiotemporal inputs [30], suitable for modeling the evolution of road distress. Recent advancements have also led to more sophisticated models [31] that reflect the probabilistic and dynamic nature of road deterioration, integrating both long-term trends and short-term fluctuations [32] observed in periodic inspection data. Short-term dynamics (e.g., extreme weather events or transient traffic overloading) may trigger rapid distress initiation or accelerated deterioration, while long-term trends govern the cumulative progression and interaction of previously developed distresses over time. However, its effectiveness is often constrained by the quality of training datasets and the complexity of road systems. Road systems are inherently complex, exhibiting highly nonstationary spatiotemporal patterns influenced by heterogeneous traffic loads, environmental conditions, and material aging. As a result, datasets used for training purely data-driven models often fail to fully capture the intricate relationships between system variables.

Recent studies in data-driven modeling have introduced several promising directions. Digital twin (DT) technologies are increasingly being adopted in road management, enabling the integration of real-time sensor data, historical records, and predictive models to support proactive maintenance and decision-making [33]. Furthermore, multimodal data fusion techniques have been increasingly explored to integrate heterogeneous data sources, such as sensor measurements, imaging data, and environmental variables, enabling more accurate and fine-grained road condition assessment [34]. Diffusion-based generative models, particularly conditional diffusion frameworks, have demonstrated strong capabilities in probabilistic spatiotemporal forecasting and uncertainty representation [35]. However, these methods still face challenges in interpretability and generalization. They fail to explicitly incorporate the underlying physical mechanisms and causal inference [36] governing the spatiotemporal evolution of road distress, such as localized diffusion and constraint effects [37]. Their black-box nature makes it difficult to explain the causal links between environmental conditions, traffic loading, and distress evolution, while purely data-driven learning may lead to spurious correlations under changing climate or traffic patterns. The lack of physics-informed consistency also constrains both the interpretability of the models and their adaptability for real-time decision support or simulation-based scenario analysis. Even when data are abundant, unconstrained ML models frequently offer limited interpretability, poor generalization to unseen scenarios, and low robustness under rare events or interventions [38]. Therefore, mechanism-informed constraints are needed to guide data-driven models toward more physically consistent and robust representations.

Multifidelity modeling [39] has emerged as a promising strategy to mitigate these limitations within a DT framework. This approach makes it possible to leverage low-fidelity (LF) data, such as high-resolution microscopic simulations of road deterioration, together with high-fidelity (HF) observations from real-world road networks. Within DT modeling, LF data enable the model to gain a preliminary understanding at reduced computational cost, while HF data continuously refine and calibrate the digital representation through feedback from the physical asset. By coupling multifidelity learning with the DT loop, the framework enhances both prediction accuracy and generalization capability while maintaining a sustainable connection between virtual and physical systems [40]. Applications of multifidelity modeling generally fall into 2 categories: approximating system responses under new or partially known state variables and enhancing model predictions for practical deployment. In the former, LF data reflect well-understood variations, whereas HF data represent responses to new, unknown, or costly-to-measure conditions [41,42]. In the latter, LF and HF data may be derived from coarse- and fine-resolution simulations [43], or analytical solutions and experiments, with HF data guiding model correction or residual learning [44].

In addition, physics-informed learning [45] has been increasingly adopted to embed prior knowledge, such as initial and boundary conditions, governing partial differential equations (PDEs) [46], and empirical relationships, directly into the ML training process [47]. This enables ML models to respect underlying physical constraints, narrow the solution space, and accelerate convergence [48,49]. However, prior studies have largely focused on constructing complex multifidelity systems, often neglecting scalability [50], particularly for road-network-scale applications. Existing approaches also typically address either local interactions or system-level approximations, without explicitly modeling the combined effects of discrete local transitions, continuous stochastic evolution, and exogenous disturbances, such as repair interventions or extreme weather events. Consequently, current models are limited in their ability to capture the dynamic interplay between microscopic processes and macroscopic patterns, reducing their applicability for real-time monitoring, scenario analysis, or simulation-based evaluation.

To address these challenges, this paper proposes a physics-embedded diffusion (PED) model to simulate the spatiotemporal evolution of road distress under natural degradation and maintenance interventions, which unifies stochastic diffusion-based [51] modeling with discrete cellular automata and disturbance-aware modeling. The PED model implicitly integrates physical knowledge into the learning process and allows simulation of interventions and extreme events through a conditional disturbance mechanism, ensuring that both local interactions and global propagation are captured within a single mathematically consistent system. This study makes the following key contributions:

• A DT model is developed for road distress evolution that enables short-term prediction of distress fluctuations arising from both intrinsic deterioration and external perturbations.
• The model captures the evolution of individual-distress types, overcoming the limitations of traditional aggregate indices (e.g., PCI) that cannot represent variations in type or severity of specific distresses.
• By integrating multifidelity learning, the framework corrects LF simulations using HF observations, enhancing extrapolation capability and ensuring physical consistency in spatiotemporal deterioration modeling across grid-level, section-level, and regional-level scales.

## Results and Discussion

### A general framework for modeling the spatiotemporal evolution of road distress

**Fig. 1.**
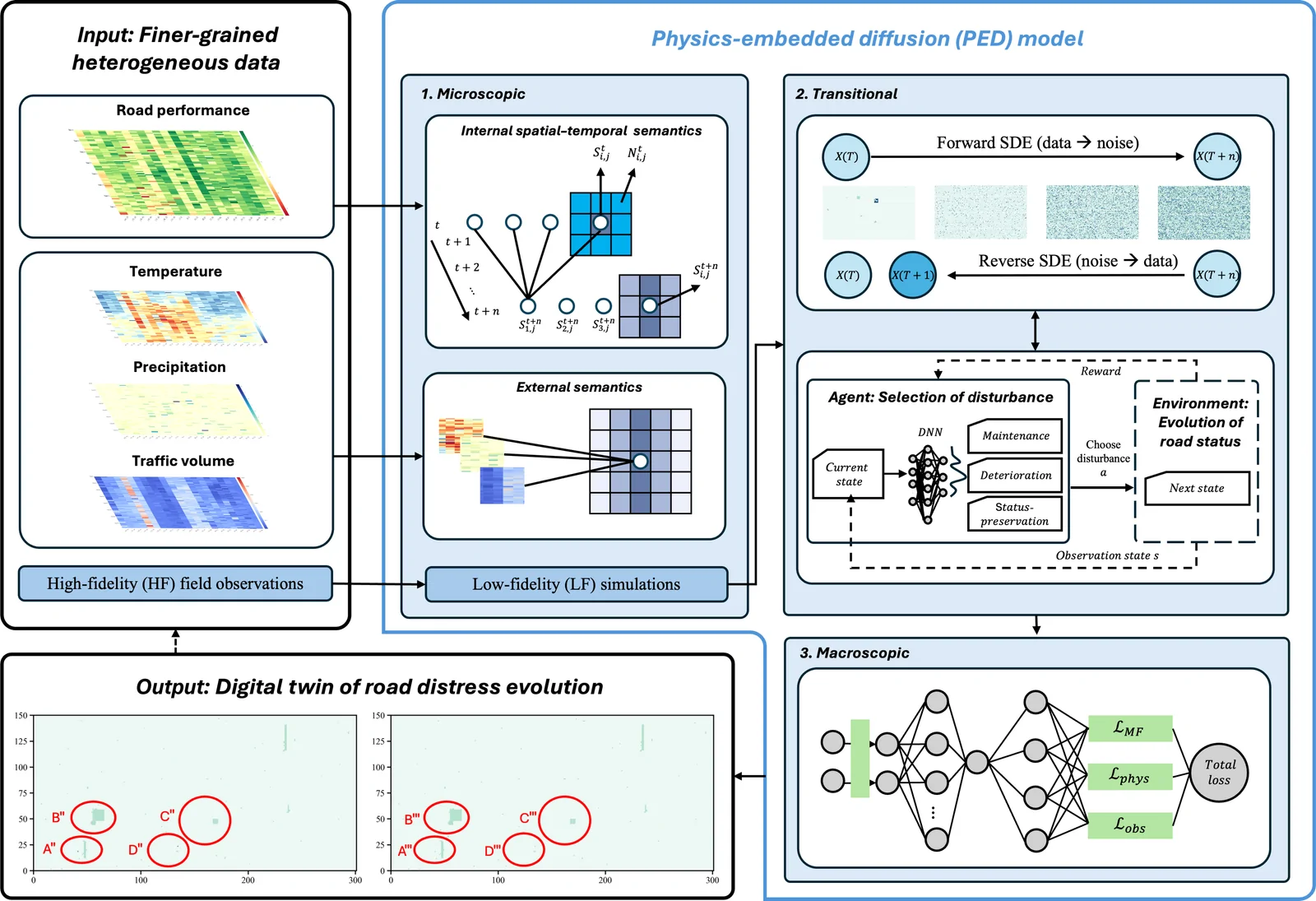
Framework of the physics-embedded diffusion (PED) model for simulating the spatiotemporal evolution of road distress.

To model the spatiotemporal evolution of road distress, the PED model integrates discrete cellular transitions, stochastic diffusion dynamics, and physics-embedded learning into a unified framework, as illustrated in Fig. 1. PED builds a continuous latent distress field $\Phi_{t}\in {\mathbb{R}}^{m\times n}$, which couples discrete local updates and global stochastic propagation through shared latent variables. This allows simultaneous representation of local transitions, global diffusion, and intervention-driven disturbance within a single mathematical structure.

The overall workflow of PED consists of 3 hierarchical layers:

• microscopic dynamics: local discrete transitions governed by neighboring states and environmental variables
• transitional diffusion: continuous stochastic propagation described by a diffusion-based generative process
• macroscopic consistency: physics-embedded constraints ensuring the model’s physical plausibility and extrapolation capability

#### Microscopic dynamics: Discrete transition module

At the microscopic level, each cell $S_{i,j}^{t}\in \left\{N,\;S_{1},\;S_{2},\;S_{3},\;S_{4},\;M\right\}$ represents a discrete distress state (e.g., cracking, pothole, or patch). Probabilistic transition rules govern the interaction between distressed cells, whether to propagate or be repaired. In the case of propagation, the nondistressed cells are likely to adopt the same type of distress as the adjacent distressed cell, simulating the spatial diffusion of distress. Conversely, in the case of repair, the affected cell transitions into a postmaintenance state, which is assumed to remain stable over short-term intervals, although long-term deterioration may still occur due to external environmental or internal factors.

To more accurately reflect real-world deterioration mechanisms, the model incorporates key external variables, including temperature, precipitation, and traffic volume. Each of these factors exerts a quantifiable influence on the probability of road surface degradation. Precipitation, for instance, is found to accelerate distress progression, particularly when exposure is sustained or occurs in high volumes. This effect often manifests with a temporal delay, indicating that moisture infiltration and weakening of sublayers may take time to translate into visible surface distress. Traffic volume also exhibits a strong positive correlation with distress severity; sections experiencing higher traffic loads tend to deteriorate more rapidly, highlighting the mechanical stress imposed by repeated vehicle passages.

The influence of environmental factors on road distress is inherently cumulative, shaped by the historical accumulation of weather conditions, traffic volumes, and past maintenance activities. These factors collectively inform the current trajectory of deterioration across the network. To capture this temporal dependency, the local transition probability is determined by the state $S_{i,j}^{t}$, neighborhood configuration $N_{i,j}^{t}$, and environmental drivers $E_{t}$, as shown in Eq. (1). It simulates fine-grained spatial interactions and captures the heterogeneity of local degradation behavior. $E_{t}$ quantifies the aggregated, time-dependent impact of past environmental at time t on the distress state. $E_{t}$ is initialized using an exponential-lag model, as shown in Eq. (2). Equation (2) assigns smaller weights to earlier time steps, thereby reflecting the varying influence of environmental factors depending on their temporal proximity. We normalize $E_{t}$ to ensure identifiability and numerical stability. Additionally, the external factor influence $I_{t-i}$ is shown in Eq. (3).

$$\begin{array}{c} P\left(S_{i,j}^{t+1}=k|S_{i,j}^{t},\;N_{i,j}^{t},\;E_{t}\right)\propto p_{k}\times \;f\left(E_{t}\right)\times \;g_{k}\left(S_{i,j}^{t},\;N_{i,j}^{t}\right) \end{array}$$ (1)

$$E_{t}=\sum_{i=0}^{n}\omega_{i}I_{t-i},\;\omega_{i}=\frac{e^{-\alpha i}}{\sum_{j=0}^{n}e^{-\alpha j}}$$ (2)

$$I_{t-i}=\frac{p_{t-i}}{p_{0}}\times \frac{N_{t-i}}{N_{0}}\times \frac{T_{t-i}}{T_{0}}$$ (3)

where k is the discrete distress state and $\omega_{i}$ denotes the impact of environmental factors $I_{t-i}$ at time t−i on the distress state, with an exponential decay function applied to assign weights for time points, where α $\left(\alpha >0\right)$ is a parameter that controls the rate of decay. Smaller values of i correspond to more recent time steps, while larger values of i represent earlier time steps. $p_{t-i}/p_{0}$ represents the variation in precipitation, where $p_{0}$ denotes the average rainfall; $N_{t-i}/N_{0}$ reflects the increase in traffic volume, where $N_{0}$ is the average traffic volume; and $T_{t-i}/T_{0}$ represents the temperature variation, where $T_{0}$ serves as the baseline average temperature. Short-term dynamics are captured through their immediate influence on recent observations $I_{t-i}$, which subsequently contribute to the environmental representation $E_{t}$. This, in turn, affects the transition probability of an individual cell, $P\left(S_{i,j}^{t+1}=k|S_{i,j}^{t},N_{i,j}^{t},E_{t}\right)$. Long-term trends are modeled through the accumulation of historical environmental information using the weighted formulation.

During the evolution process, the deterioration probability $p_{e}$, the maintenance probability $p_{m}$, and the probability of status preservation $p_{u}$ satisfy the equation $p_{e}+p_{m}+p_{u}=1$. For each distress cell, the maintenance probability $p_{m}$ was first evaluated, followed by the deterioration probability $p_{e}$ conditional on no maintenance occurring. The remaining probability was assigned to the status-preserving state $p_{u}$. To better simulate realistic recovery and deterioration dynamics, a cooling mechanism was introduced into the cellular evolution model, whereby repaired cells enter a temporary inactive state recorded in a cooldown map before they can deteriorate again. To enable coupling with the continuous diffusion layer, each discrete state is embedded into a continuous scalar value through a mapping, as shown in Eq. (4):

$$\Phi_{i,j}^{t}=\varepsilon \left(S_{i,j}^{t}\right)$$ (4)

where $\varepsilon \left(\cdot \right)$ is a differentiable embedding operator that preserves topological proximity between adjacent distress levels.

Therefore, at each discrete time step t, the model maintains 2 synchronized representations of the system state:

• a continuous distress field $\Phi_{t}\in {\mathbb{R}}^{m\times n}$, representing the physical deterioration level (e.g., PCI)
• a discrete distress field $S_{t}={\left\{N,\;S_{1},\;S_{2},\;S_{3},\;S_{4},\;M\right\}}^{m\times n}$

#### Transitional diffusion: Continuous diffusion-based generation module

The stochastic evolution of the continuous distress field $\Phi_{t}\in {\mathbb{R}}^{m\times n}$ is governed by a physics-embedded PDE that integrates both diffusion and action-driven transitions, as shown in Eq. (5):

$$\begin{array}{c} \Phi^{t+1}=\Phi^{t}+D\nabla^{2}\Phi^{t}+f_{\text{local}}\left(\Phi^{t},\;N^{t},\;P,\;E_{t}\right)+A\left(s,\;a,\;t\right)+\xi_{t} \end{array}$$ (5)

where $D\nabla^{2}\Phi$ represents spatiotemporal diffusion, capturing the continuous spread of distress, and $f_{\text{local}}\left(\Phi^{t},\;N^{t},\;P,\;E_{t}\right)$ encodes localized rule-based transitions, dependent on neighboring states N, parameter set P, and environmental indicator $E_{t}$. $A\left(s,\;a,\;t\right)$ is a disturbance-aware source term, embedding the influence of deterioration, maintenance, or status-preserving decisions into the evolution, and $\xi_{t}$ denotes stochastic perturbations modeling environmental noise.

Equation (5) achieves a mathematically consistent coupling of discrete (local) and continuous (global) mechanisms, allowing the model to express both localized deterioration propagation and global stochastic diffusion within one framework. At each time step, a network $\pi_{\psi }\left(a_{t}|s_{t}\right)$ generates disturbances, and their effects are immediately reflected in the diffusion equation through $A\left(s,\;a,\;t\right)$. This forms a closed-loop system, as shown in Eq. (6):

$$s_{t}\overset{\pi_{\psi }}{\to }a_{t}\overset{\mathrm{PED}\;\text{dynamics}}{\to }s_{t+1}$$ (6)

This end-to-end disturbance mechanism enables the simulation of interventions and extreme events within a single framework, ensuring both physical interpretability and model stability.

The final continuous field $\Phi_{t+1}$ is updated by combining the denoised prediction ${\tilde{\Phi }}_{t+1}$ with local Laplacian diffusion, sparse stochastic propagation, and disturbance-aware source term injection, as shown in Eq. (7). Finally, the continuous field $\Phi_{t+1}$ is discretized to the discrete state $S_{t+1}={\left\{N,\;S_{1},\;S_{2},\;S_{3},\;S_{4},\;M\right\}}^{m\times n}$ using a piecewise threshold function $Q\left(\cdot \right)$, as shown in Eq. (8).

$$\begin{array}{c} \Phi_{t+1}={\tilde{\Phi }}_{t+1}+D\cdot \left(K*{\tilde{\Phi }}_{t+1}\right)+A\left(a_{t}\right)⊙M_{s}+\eta_{t} \end{array}$$ (7)

$$S_{i,j}^{t+1}=Q\left(\Phi_{i,j}^{t+1}\right)=c,\text{if}\;\Phi_{i,j}^{t+1}\in \left[{ℸ}_{k-1},{ℸ}_{k}\right)$$ (8)

where K is the discrete Laplacian kernel. This simple kernel provides a physically interpretable mechanism for local spreading and can be scaled by the diffusion coefficient D>0 to adjust interaction strength. $A\left(a_{t}\right)$ is the action-conditioned source term encoding interventions (e.g., maintenance), $M_{s}\in {\left\{0,1\right\}}^{m\times n}$ is a stochastic sparsity mask (e.g., 30% of active cells are updated), ⊙ denotes element-wise multiplication, and $\eta_{t}$ is a small additive noise term to account for unobserved stochasticity. Thresholds $\left\{{ℸ}_{0},{ℸ}_{1},\ldots ,{ℸ}_{c}\right\}$ are determined via clustering or empirical calibration.

#### Macroscopic consistency: Physics-embedded multifidelity coupling module

At the macroscopic level, PED integrates multifidelity learning to enhance generalization. The LF simulation outputs, representing microscopic dynamics and transitional diffusion, are constrained by HF field observations and environmental data. This coupling is achieved through a physics-embedded neural network that simultaneously minimizes the governing PDE residuals and learns the LF–HF discrepancy. PED embeds physics logic within a continuous–discrete hybrid field, enabling end-to-end simulation while maintaining mechanistic plausibility and strong generalization beyond the training regime. The total loss (Eq. (9)) combines the fidelity loss $L_{\mathrm{MF}}$, physics-based loss $L_{\text{phys}}$, and observation loss $L_{\mathrm{obs}}$ (Eqs. (10) to (12)), which respectively capture the LF–HF consistency, enforce physical mapping, and align predictions with real measurements.

$$L_{\text{total}}=\lambda_{\mathrm{MF}}L_{\mathrm{MF}}+\lambda_{\text{phys}}L_{\text{phys}}+\lambda_{\mathrm{obs}}L_{\mathrm{obs}}$$ (9)

where $\lambda_{\mathrm{MF}}$, $\lambda_{\text{phys}}$, and $\lambda_{\mathrm{obs}}$ are the weighting coefficients assigned to the corresponding loss components.

The multifidelity consistency loss is defined as

$$\begin{array}{c} L_{\mathrm{MF}}=\frac{1}{N_{\mathrm{HF}}}\sum_{i=1}^{N_{\mathrm{HF}}}{\left|u^{\mathrm{LF}}\left(x_{i},\;t_{i}\right)+\Delta u_{\theta }\left(x_{i},\;t_{i}\right)-u_{i}^{\mathrm{HF}}\right|}^{2} \end{array}$$ (10)

where $u^{\mathrm{LF}}\left(x_{i},\;t_{i}\right)$ denotes the LF simulated response, $u_{i}^{\mathrm{HF}}$ denotes the corresponding HF observation, and $\Delta u_{\theta }\left(x_{i},\;t_{i}\right)$ is the correction term predicted by the network. This loss constrains the corrected LF prediction to remain consistent with the available HF data.

The physics-consistency loss is expressed as

$$\begin{array}{c} L_{\text{phys}}=\frac{1}{N_{\text{phys}}}\sum_{i=1}^{N_{\text{phys}}}{\left|\Phi_{\theta }^{t_{i}+1}\left(x_{i}\right)-T\left(\Phi_{\theta }^{t_{i}}\left(x_{i}\right),\;N^{t_{i}},\;E^{t_{i}},\;P^{t_{i}},\;A^{t_{i}}\right)\right|}^{2} \end{array}$$ (11)

where $\Phi_{\theta }^{t_{i}}\left(x_{i}\right)$ is the predicted distress state at location $x_{i}$ and time $t_{i}$ and $T\left(\cdot \right)$ denotes the physics-based state-transition operator. The variables $N^{t_{i}}$, $E^{t_{i}}$, $P^{t_{i}}$, and $A^{t_{i}}$ represent the neighborhood state, environmental conditions, physical parameters, and action or intervention variables, respectively. $N_{\text{phys}}$ is the number of samples used to evaluate the physical consistency constraint.

The observation loss is given by

$$\begin{array}{c} L_{\mathrm{obs}}=\frac{1}{N_{\mathrm{obs}}}\sum_{i=1}^{N_{\mathrm{obs}}}{\left|\Phi_{\theta }\left(x_{i},\;t_{i}\right)-\Phi^{\mathrm{obs}}\left(x_{i},\;t_{i}\right)\right|}^{2} \end{array}$$ (12)

where $\Phi^{\mathrm{obs}}\left(x_{i},\;t_{i}\right)$ denotes the observed distress state and $N_{\mathrm{obs}}$ is the number of available observations. This term directly minimizes the discrepancy between model predictions and field measurements.

$L_{\mathrm{obs}}$ is implicitly formulated to jointly evaluate PCI prediction accuracy and weighted distress-area consistency across different distress categories. Specifically, PCI prediction accuracy is estimated according to the Highway Performance Assessment Standards (JTG 5210-2018) [52], in which longitudinal cracking and transverse cracking are assigned weighting factors of 2.0, alligator cracking and potholes are assigned weighting factors of 1.0, and repaired or patched areas are assigned a lower weighting factor of 0.1. Meanwhile, weighted distress-area accuracy is quantified using the mean weighted percentage error of area (area MWPE) metric defined in Eq. (13), where the weighting factors for different distress categories are kept consistent with those adopted in the PCI evaluation. This weighting strategy enables the reward mechanism to better capture the practical contribution of different distress categories to overall road condition deterioration.

$$\begin{array}{c} \text{Area MWPE}\;\left(\%\right)=\frac{\sum_{k}w_{k}\times \frac{\left|A_{k}^{\text{pred}}-A_{k}^{\text{true}}\right|}{\text{max}\left(A_{0},\;A_{k}^{\text{true}}\right)}}{\sum_{k}w_{k}}\times 100 \end{array}$$ (13)

where $w_{k}$ is the weighting coefficient associated with the kth distress category. $A_{k}^{\text{pred}}$ denotes the predicted distress area for category k, $A_{k}^{\text{true}}$ denotes the ground-truth distress area for category k, and $A_{0}$ is a baseline area threshold introduced to avoid excessively amplified percentage errors when the true distress area is very small. Each computational cell primarily represents a discrete road condition state and its spatial location, rather than indicating that the entire geometric footprint of the cell is physically affected by distress. The observed distress area is estimated by converting the number of cells assigned to each distress category using the corresponding equivalent-area mapping coefficients. This distinction allows the model to preserve a computationally manageable spatial representation while maintaining consistency with the measured distress areas.

The 3 loss components correspond to different aspects of model performance. $L_{\mathrm{MF}}$ primarily measures the consistency between the LF and HF representations, $L_{\text{phys}}$ reflects compliance with the prescribed physical transition process and corresponds to the physical-loss values reported during training, and $L_{\mathrm{obs}}$ is directly associated with the prediction errors evaluated using the PCI-based metrics and area MWPE. These loss terms are optimization objectives rather than evaluation indices themselves; the reported evaluation metrics are calculated independently from the model outputs. Together, the 3 components enable the PED framework to integrate multifidelity consistency, physical plausibility, and agreement with observations within a unified continuous–discrete deterioration field.

### Influence of hyperparameter selection on model performance

To identify the optimal hyperparameter configuration for the proposed PED model, we conducted a comprehensive grid search across 5 key parameters:

• Learning rate η of the Adam optimizer (0.0001, 0.0005, and 0.001), which governs the step size of gradient updates for the model and directly affects the stability and convergence rate.
• Discount factor γ (0.98, 0.99, and 0.995), which controls the weighting of future rewards in the model’s update rule, with larger values emphasizing long-term dynamics in distress evolution, while a smaller value prioritizes immediate feedback.
• Epsilon-decay rate $\epsilon_{d}$ (0.98, 0.99, and 0.995), which determines the exploration schedule during training, with values closer to 1 maintaining exploration for longer periods, while smaller values lead to faster convergence into a greedy policy.
• Physical-loss weight ℯ (0.2, 0.3, and 0.5), which regulates the contribution of the physics-informed residual term to the total loss, enabling the model to balance data-driven learning and physical consistency. The physical loss initially exhibited a much smaller magnitude than the other loss components and was scaled to a comparable level.
• Diffusion rate π (0.008, 0.02, and 0.05), which controls the strength of Laplacian-based smoothing applied during distress propagation, with higher values yielding stronger regularization and lower values enhancing responsiveness to local variations.

A total of 3^5^ = 243 experiments were carried out to explore all possible hyperparameter combinations. The experiments were conducted on the China Fine-grained Road Distress Dataset (FRD-CN) (see Methods).

Prior to simulation, all other parameters and constants of the PED model were carefully initialized. The physical road domain considered in the FRD-CN case study is 15 m wide and 160 km long. It was represented using a 2-dimensional computational grid comprising 150 cells across the road width and 1,600 cells along the longitudinal direction. Accordingly, one grid row corresponds to 0.1 m in the transverse direction, while one grid column corresponds to 0.1 km (100 m) in the longitudinal direction. The full road was further divided into 32 spatial sectors, each covering 5 km and corresponding to 50 longitudinal grid columns. Considering the computational cost of training, we selected 25% of the dataset under initial setup to conduct the hyperparameter evaluation experiments. A total of 265 iterations were performed per simulation to ensure temporal convergence, with each iteration representing 1 d. The degradation probabilities were initialized as $p_{e1}=0.1$ for cracks and $p_{e2}=0.075$ for potholes based on study of typical cases in the data. Longitudinal cracks spread along the road’s length, transverse cracks across its width, and alligator cracks diagonally through interconnected patterns, and potholes expand outward in all directions. Precipitation, traffic volume, and temperature were incorporated into the model through a relative environmental influence factor rather than as fixed absolute inputs. Following normalization and temporal aggregation, the 3 factors were combined and scaled against their global mean, producing a sector-specific coefficient that dynamically adjusted road distress propagation and repair probabilities. In addition, min–max normalization was applied to ensure that the magnitudes of loss components are comparable during training. Model performance was assessed using the area MWPE index.

**Fig. 2.**
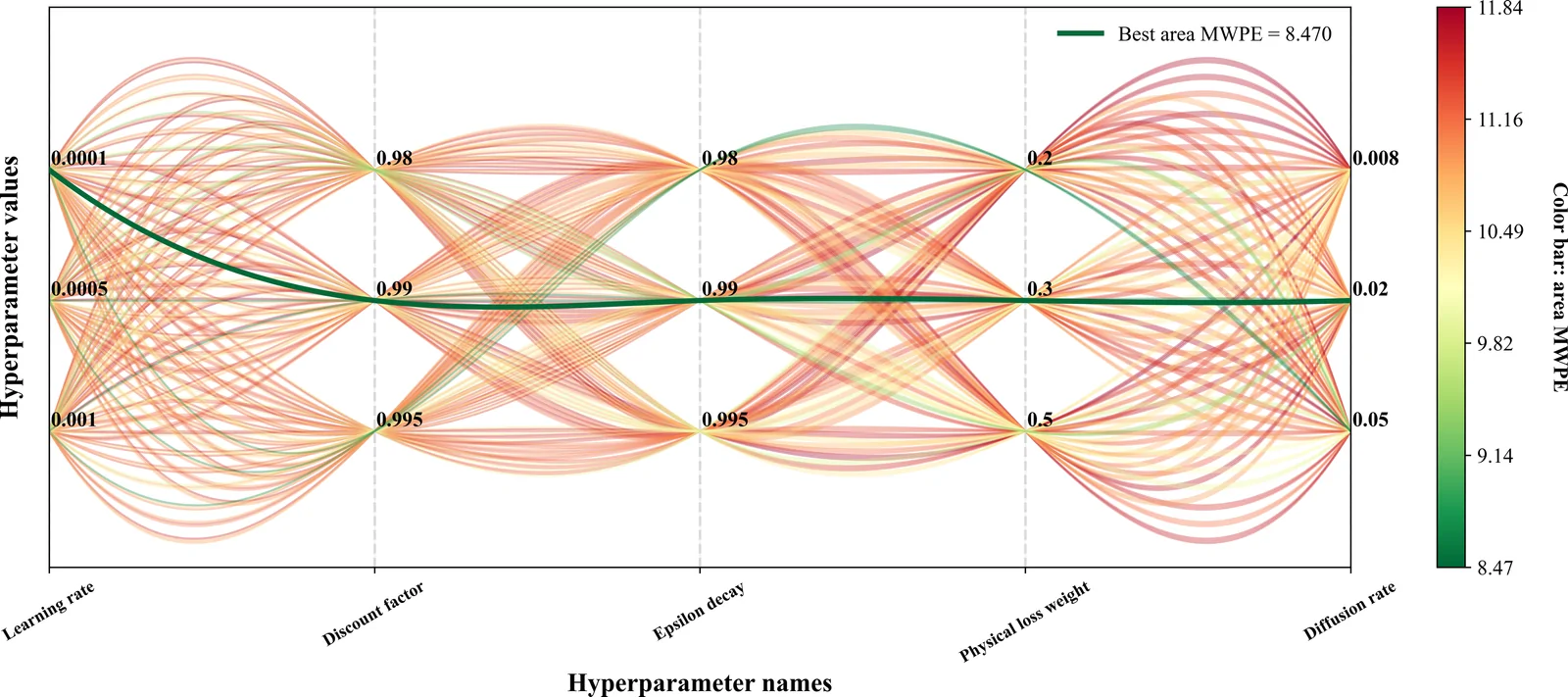
Parallel coordinates of hyperparameter combinations colored by mean weighted percentage error of area (area MWPE). Each vertical axis represents one type of hyperparameter, while vertical positions denote the parameter values, and each curve corresponds to one configuration. The line color encodes the resulting area MWPE (green = lower error), with the best-performing configuration highlighted in darkest green.

The parallel-coordinates visualization in Fig. 2 reveals that the lower learning rate η (0.0001) slightly improves stability, while a medium discount factor γ (0.99) enhances long-term consistency. Similarly, medium epsilon decay ($\epsilon_{d}=0.99$) maintains exploration longer and achieves lower errors. The physical-loss weight ℯ shows an optimal range around 0.3; too small weakens physical constraints, and too large limits flexibility. The moderate diffusion rate π (0.02) improves spatial smoothness and predictive accuracy. In summary, the best configuration features a lower learning rate (0.0001); moderate discount factor (0.99), epsilon decay (0.99), and physical-loss weight (0.3); and relatively strong diffusion (0.02), underscoring the need to balance exploration, long-term reward guidance, physical consistency, and diffusion-driven stabilization for optimal performance.

### Influence of environmental history window length and decay rate

To determine the optimal environmental history window length, we conducted comparative experiments under same initialization settings and fixed optimal hyperparameters from the prior evaluation. Model performance was assessed using area MWPE, PCI root mean square error (PCI RMSE), PCI mean absolute error (PCI MAE), and PCI mean absolute percentage error (PCI MAPE). The results are summarized in Table 1.

**Table 1.** Performance comparison across environmental history window lengths. Bold—the best performance model; with an initial decay rate fixed at 0.5.

Window length	Predicted indicators/mean (min, max)			
	Area MWPE/%	PCI RMSE	PCI MAE	PCI MAPE/%
30	4.391 (0.015, 16.486)	10.555 (7.070, 15.396)	8.573 (5.454, 13.028)	9.298 (5.774, 14.515)
40	5.001 (0.015, 16.356)	10.523 (7.070, 15.448)	8.538 (5.454, 13.069)	9.263 (5.774, 14.556)
45	4.795 (0.002, 16.316)	10.519 (7.070, 15.498)	8.545 (5.454, 12.991)	9.270 (5.774, 14.478)
**50**	**4.484** **(0.015, 16.213)**	**10.526** **(7.070, 15.487)**	**8.537** **(5.454, 12.974)**	**9.262** **(5.774, 14.460)**
55	5.174 (0.022, 16.947)	10.584 (7.140, 15.507)	8.618 (5.518, 13.188)	9.344 (5.839, 14.675)
60	4.919 (0.030, 16.815)	10.576 (7.111, 15.561)	8.598 (5.490, 13.192)	9.323 (5.811, 14.680)
70	4.825 (0.004, 16.176)	10.564 (7.144, 15.511)	8.576 (5.514, 12.997)	9.301 (5.835, 14.484)

Area MWPE, mean weighted percentage error of area; PCI, Pavement Condition Index; RMSE, root mean square error; MAE, mean absolute error; MAPE, mean absolute percentage error

The results showed that shorter windows (30) achieved reasonable accuracy in area MWPE (=4.391%) but exhibited higher variability in PCI metrics (PCI MAPE = 9.298%). A length of 50 consistently yielded the best results, with the lowest PCI MAPE (9.262%), indicating strong agreement with observed deterioration and modest spatial consistency (area MWPE = 4.484%). Longer windows (55 to 70) offered no further gains and occasionally increased uncertainty, suggesting redundant information.

To assess the influence of historical feature decay, additional experiments were performed with the window fixed at 50 and decay rates ranging from 0.3 to 0.7. The performance metrics are summarized in Table 2.

**Table 2.** Performance comparison across decay rate of the historical environmental features. Bold—the best performance model.

Decay rate	Predicted indicators/mean (min, max)			
	Area MWPE/%	PCI RMSE	PCI MAE	PCI MAPE/%
0.3	4.834 (0.025, 16.348)	10.512 (7.070, 15.341)	8.529 (5.454, 13.061)	9.254 (5.774, 14.547)
0.4	4.677 (0.008, 16.491)	10.531 (7.070, 15.401)	8.538 (5.454, 13.043)	9.263 (5.774, 14.531)
**0.5**	**4.484** **(0.015, 16.213)**	**10.526** **(7.070, 15.487)**	**8.537** **(5.454, 12.974)**	**9.262** **(5.774, 14.460)**
0.6	4.937 (0.026, 15.535)	10.529 (7.111, 15.309)	8.537 (5.490, 12.896)	9.262 (5.811, 14.382)
0.7	4.846 (0.004, 16.651)	10.573 (7.144, 15.422)	8.586 (5.514, 13.074)	9.311 (5.835, 14.561)

Area MWPE, mean weighted percentage error of area; PCI, Pavement Condition Index; RMSE, root mean square error; MAE, mean absolute error; MAPE, mean absolute percentage error

Area MWPE demonstrated moderate sensitivity to variations in the decay rate. A decay rate of 0.5 achieved the lowest area MWPE (4.484%), reflecting balanced accuracy across scales. Higher rates (0.6 to 0.7) produced higher errors, indicating that excessive retention or rapid forgetting of historical effects reduces accuracy. Lower rates (0.3) caused higher area MWPE as well. Therefore, a decay rate of 0.5 provides the optimal trade-off, capturing meaningful environmental history without overemphasizing outdated information.

### Ablation study and model performance comparison

To evaluate the effectiveness of each component in our proposed framework, ablation studies were also conducted on both FRD-CN and the US Long-Term Pavement Performance Dataset (LTPP-US). The FRD-CN ablation study adopted the same road-grid configuration described above. For the LTPP-US ablation study, a 150 × 1,800-cell grid was used, with the road sections grouped into 36 spatial regions according to their geographic coordinates.

The experiments used the same initialization settings and fixed optimal hyperparameters, testing 5 model variants with different core module combinations: (a) full model, (b) no transitional diffusion, (c) no physics-embedded multifidelity constraint, (d) no disturbance-aware module, and (e) no external environmental inputs.

**Fig. 3.**
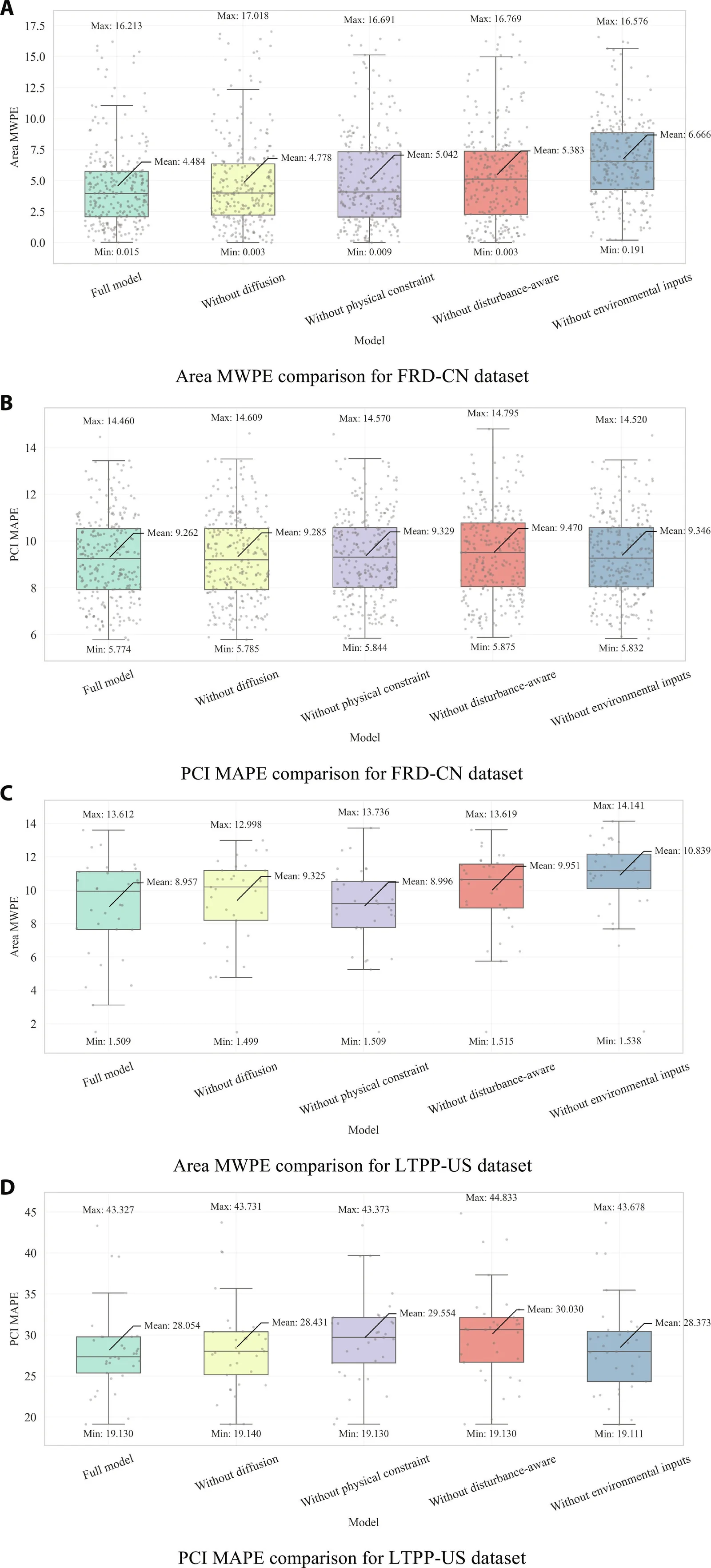
Comparison of model performance in ablation studies. (A) Mean weighted percentage error of area (area MWPE) comparison for the China Fine-grained Road Distress Dataset (FRD-CN) dataset. (B) Pavement Condition Index (PCI) mean absolute percentage error (MAPE) comparison for the FRD-CN dataset. (C) Area MWPE comparison for the US Long-Term Pavement Performance Dataset (LTPP-US) dataset. (D) PCI MAPE comparison for the LTPP-US dataset.

The FRD-CN and LTPP-US ablation models were trained and evaluated for 265 and 33 time steps, respectively. Prediction accuracy was measured using area MWPE and PCI MAPE. Figure 3 illustrates the error distributions across time steps using boxplots and scatter points, with the horizontal axis denoting the 5 model variants and the vertical axis showing error values, including mean, maximum, and minimum statistics.

As shown in Fig. 3A, the full model yielded the lowest mean area MWPE error (4.484%), whereas excluding disturbance-aware (5.383%) or environmental inputs (6.666%) caused the largest degradation. The diffusion-removed variant has a slightly higher mean area MWPE (4.778%), suggesting that diffusion improves area-level estimation. The physical-constraint-removed model also yields higher error (5.042%) and variance, underscoring the importance of physical constraints.

A similar pattern appears in PCI MAPE (Fig. 3B): The full model achieves the lowest prediction error, with a PCI MAPE of 9.262%. Removing the diffusion module slightly increases the error to 9.285%, while excluding the physics-informed constraint results in a further increase to 9.329%. Although these differences are modest, the consistently lower error of the full model indicates that both mechanisms contribute to improved predictive accuracy. The diffusion module helps represent the spatial propagation and neighborhood-dependent evolution of road distress, whereas the physics-informed constraint regularizes the deterioration process by encouraging predictions to remain consistent with the underlying physical evolution pattern. Their combined use therefore improves the model’s ability to capture both local distress interactions and the overall temporal deterioration trend. The variants without the disturbance-aware mechanism and without environmental inputs yield the highest PCI MAPE values, at 9.470% and 9.346%, respectively. This result highlights the importance of incorporating both maintenance-related disturbances and environmental conditions to improve the model’s predictive performance.

Consistent trends were found on the LTPP-US dataset (Fig. 3C and D). The full model again achieves the best PCI prediction (mean MAPE = 28.054%) with removing diffusion (28.431%), while removing the physical constraint (29.554%), disturbance (30.030%), or environmental input (28.373%) modules significantly degrades performance. In area MWPE, the full model (8.957%) outperforms all variants, with the environmental-input-removed model showing the largest deterioration (10.839%). The stronger sensitivity to environmental information removal in the LTPP-US dataset suggests that environmental factors become more influential in long-term forecasting.

Overall, both datasets indicate that the full model achieves an optimal balance between global PCI prediction accuracy and fine-grained distress-level prediction performance. The cross-dataset comparison also demonstrates strong generalization across regions and time scales (short- and long-term), although performance on LTPP-US is lower due to its coarser temporal granularity and greater heterogeneity. These findings also highlight that fine-grained data substantially enhance model accuracy and reliability.

**Fig. 4.**
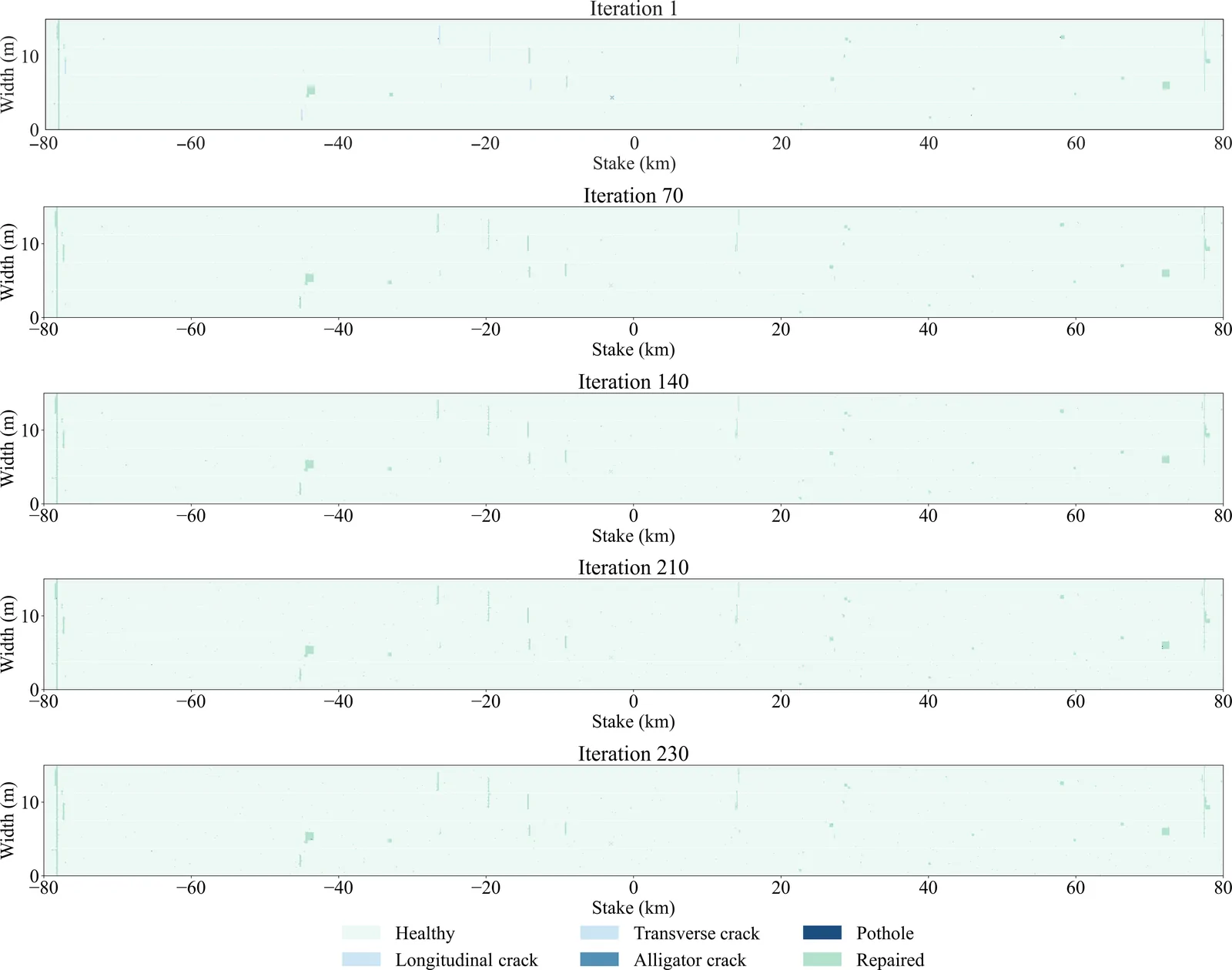
Spatiotemporal evolution of road distress in time steps.

To further illustrate the simulation results, we visualize the spatiotemporal evolution of road conditions corresponding to the FRD-CN dataset. In Fig. 4, the horizontal axis represents the longitudinal direction of the road, and the vertical axis corresponds to the lane width. Four lanes are delineated by white dashed lines. For each cell, light green represents a healthy state; shades of blue (from light to dark) denote longitudinal, transverse, and alligator cracks and potholes as shown in the legend; and dark green indicates repaired areas. The figures present 4 representative snapshots: the initial condition and the simulated states after 70, 140, 210, and 230 time steps.

It can be observed that crack patterns progressively intensified over time. Alligator cracking developed severely compared with other crack types, with noticeable deterioration emerging around stake number −5. Sudden pothole initiations are observed near stake −20 around iteration 70 (approximately 2022 August 3). This rapid initiation appears to be associated with short-term dynamics, as a localized rainfall peak can be identified around the same period (see the “Road distress data source” section in Methods), which may have accelerated the deterioration process. From a long-term perspective, this event coincides with a period of rising temperatures beginning in July and reaching a peak around August, along with sustained rainfall during July and early August. Although these rainfall events are not the most intense, their persistence may have contributed to cumulative moisture-related damage, facilitating the observed distress evolution. Similarly, around iteration 230 (approximately 2023 January 10), new potholes are observed near stake +25. This event also appears to be associated with a short-term rainfall peak around that date. In addition, longer-term conditions, including gradually decreasing temperatures during winter and relatively low precipitation in the surrounding period, may have contributed to material stiffening and increased vulnerability to traffic-induced damage. Pothole regions have undergone rapid repair. Nevertheless, even after these repairs, areas of previously distressed areas might still exhibit renewed signs of distress (e.g., around stake numbers −45 and +72), which was highly consistent with real-world deterioration pattern.

At the section level, several consistent patterns can also be observed. First, transverse cracking within the region tends to exhibit more severe deterioration (into potholes) during winter periods (around stake −80), which is likely associated with temperature drops that induce material contraction and increase tensile stress. Second, cracks tend to extend more frequently during both hot-humid periods (summer, around iteration 70) and cold-wet periods (winter, around iteration 230). In summer, higher humidity and sustained rainfall may promote moisture infiltration and cumulative distress, while in winter, lower temperatures can lead to increased stiffness and brittleness of the asphalt binder, making it more susceptible to aggregate loss under traffic loading. In addition, larger diurnal temperature variations in both seasons may induce repeated thermal expansion–contraction cycles, contributing to fatigue accumulation and crack propagation.

**Fig. 5.**
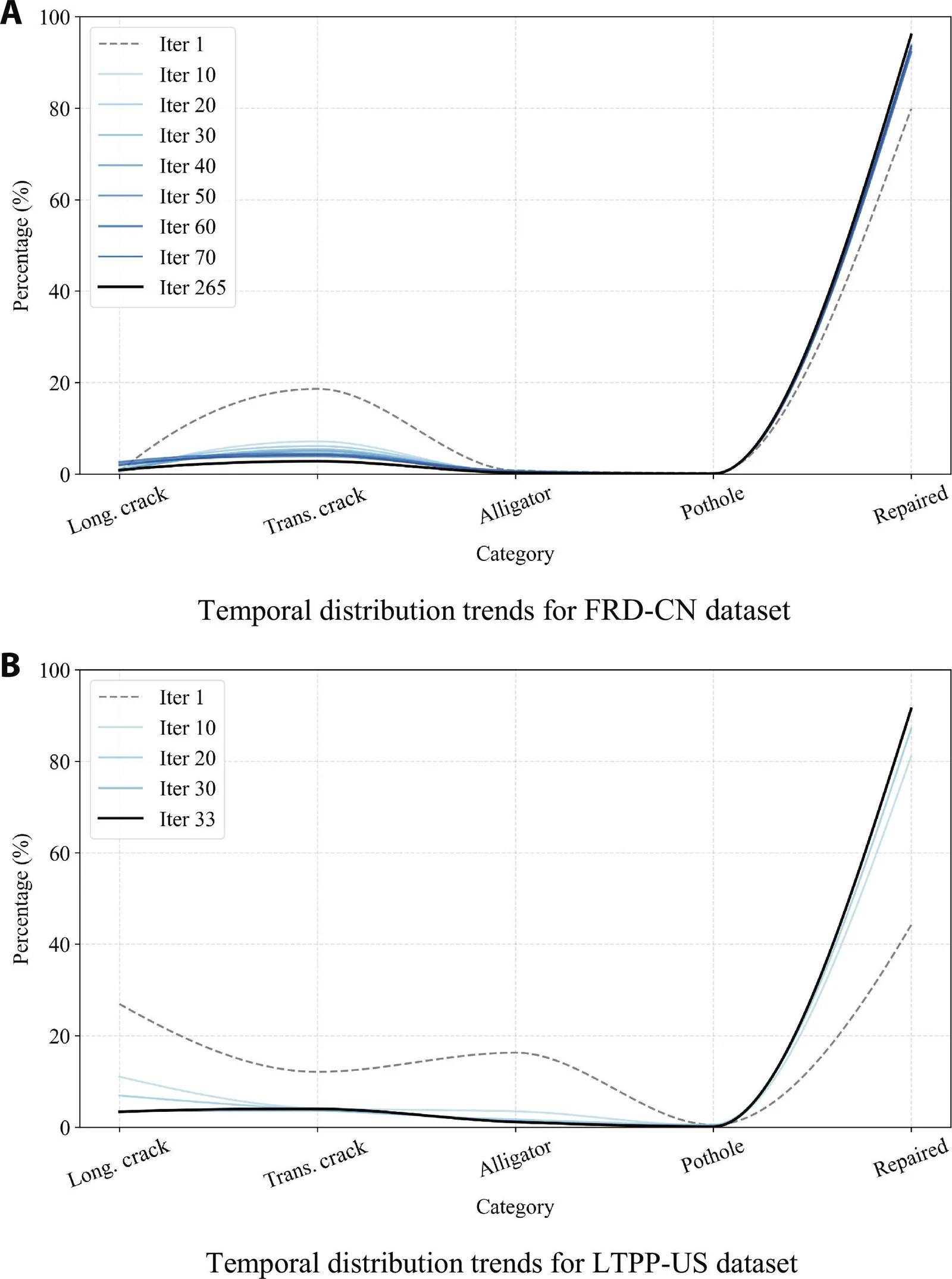
Temporal distribution trends of different distress types. (A) Temporal distribution trends for the China Fine-grained Road Distress Dataset (FRD-CN) dataset. (B) Temporal distribution trends for the US Long-Term Pavement Performance Dataset (LTPP-US) dataset.

In addition, Fig. 5 illustrates the temporal evolution of different distress types throughout the simulation. The horizontal axis represents distress categories, while the vertical axis shows their corresponding proportions. Blue curves, deepening in color with increasing iteration steps, depict the progression over time. Initially, various types of distress were distributed across the road section. In the FRD-CN dataset (Fig. 5A), the proportions of longitudinal and transverse cracks gradually decreased, and alligator cracking also diminished. Potholes had a relatively small proportion. The LTPP-US dataset (Fig. 5B) exhibited a similar pattern. As observation years progressed, longitudinal, transverse and alligator cracking repeatedly exhibited a pattern of decrease. The proportion of potholes remained consistently low, likely due to effective preventive maintenance, routine resurfacing, and strict safety standards in US highway networks. The extent of repairs showed a rise that stabilized at a high level, further evidencing the sustained effectiveness of maintenance practices.

### Model performance comparison across state-of-the-art models

We conducted a performance comparison between the proposed model and state-of-the-art models. The state-of-the-art models include the PED model enhanced with a neural PDE evolver, and 4 advanced physics-informed neural network (PINN) variants: (a) finite-difference PINN (fPINN), (b) residual-based PINN, (c) Fourier PINN (FNN), and (d) adaptive multidomain PINN (XPINN). fPINN is designed to incorporate finite-difference discretization into the physics-informed learning framework, making it particularly suitable for approximating localized spatial variations and sharp state transitions in road deterioration. Since road surface degradation often exhibits strong local dependency and neighborhood-driven propagation behavior, the finite-difference formulation in fPINN provides a natural mechanism for modeling discrete spatial gradients and localized distress diffusion. Residual-based PINN emphasizes enforcing governing equations through residual minimization, thereby improving the physical consistency of the predicted deterioration process by constraining the solution space according to predefined evolution dynamics. FNN leverages Fourier feature representations to capture periodic and globally smooth patterns. This design is theoretically motivated by the fact that road deterioration often contains low-frequency global trends driven by long-term environmental loading, traffic accumulation, and seasonal cyclic effects. Fourier mappings enhance the network’s ability to represent multiscale spatial-frequency components and alleviate the spectral bias problem commonly observed in standard neural networks when approximating high-frequency variations. XPINN further decomposes the computational domain into multiple subregions, enabling localized physics constraints and independent subnet optimization. This mechanism is particularly suitable for road systems because road deterioration processes are highly heterogeneous across different spatial regions due to varying traffic loads, environmental exposure, and maintenance histories. Domain decomposition, therefore, improves the model’s capability to capture spatial heterogeneity and multiscale interactions within large-scale road networks. Overall, these model variants were selected because their underlying mathematical structures correspond to different physical characteristics of road deterioration, including localized diffusion behavior, global temporal evolution, spatial heterogeneity, and multiscale deterioration interactions.

All models were implemented within the original training framework and interface definitions, allowing seamless switching under the same setup to ensure experimental comparability. Evaluation was conducted using area MWPE, PCI RMSE, PCI MAE, PCI MAPE, and computing time, with the results summarized in Table 3. All computational experiments were conducted on a laptop equipped with an 8-core ARM-based processor comprising 4 performance cores and 4 efficiency cores. All models were executed in CPU-only mode without discrete GPU acceleration. The implementation was developed using Python 3.9.18 and PyTorch 2.0.0. The computation times reported in Table 3 were measured under the same hardware and software environment for all models to ensure a consistent comparison.

**Table 3.** Model performance comparison with state-of-the-art models. Bold—the best performance models. For each state-of-the-art variant, only the best-performing configuration is shown. A detailed performance comparison of all 5 variants under different parameter settings is provided in Table S1 of the Supplementary Materials.

State-of-the-art model	Predicted indicators/mean (min, max)				
	Area MWPE/%	PCI RMSE	PCI MAE	PCI MAPE/%	Computing time/s
**PED**	**4.484** **(0.015, 16.213)**	10.526 (7.070, 15.487)	8.537 (5.454, 12.974)	9.262 (5.774, 14.460)	467.17
Neural PDE	45.581 (0.649, 100.954)	**10.159** **(6.694, 15.112)**	**8.325** **(5.302, 12.777)**	**9.041** **(5.613, 14.253)**	**290.60**
fPINN (hidden dim = 128, layers = 4)	4.749 (0.012, 16.245)	10.530 (7.137, 15.322)	8.551 (5.469, 13.009)	9.276 (5.789, 14.496)	1,241.93
Residual-based PINN (hidden dim = 256, layers = 4)	4.700 (0.026, 16.227)	10.516 (7.154, 15.479)	8.525 (5.496, 13.008)	9.249 (5.817, 14.495)	6,423.39
FNN (hidden dim = 128, layers = 4)	4.874 (0.079, 17.003)	10.555 (7.154, 15.414)	8.561 (5.503, 13.120)	9.286 (5.824, 14.608)	1,428.87
XPINN (hidden dim = 256, layers = 4)	4.516 (0.015, 17.003)	10.567 (6.983, 15.515)	8.592 (5.428, 13.182)	9.317 (5.749, 14.670)	1,172.11

Area MWPE, mean weighted percentage error of area; PCI, Pavement Condition Index; RMSE, root mean square error; MAE, mean absolute error; MAPE, mean absolute percentage error; PED, physics-embedded diffusion; PDE, partial differential equation; PINN, physics-informed neural network; fPINN, finite-difference PINN; FNN, Fourier PINN; XPINN, adaptive multidomain PINN

The neural PDE model exhibited a modest improvement over the PED model in macroscopic PCI prediction, reducing the error from 9.262% to 9.041%, indicating that incorporating more complex fatigue mechanisms at the macroscopic scale enhances the physical representation of deterioration processes. However, at the individual-distress level, the error increased markedly from 4.484% to 45.581%. This discrepancy suggests that although the neural PDE formulation improves the overall smoothness and global consistency of deterioration dynamics, it may simultaneously weaken the model’s capability to preserve fine-grained local distress patterns. In particular, the diffusion-driven propagation mechanism tends to spatially redistribute distress states, which can amplify local deviations in regions with sparse or highly heterogeneous distress distributions.

Most PINN variants performed worse in both macroscopic and microscopic tasks, except residual-based PINN variant. The others’ macroscopic prediction errors (9.276%, 9.286%, and 9.317%) all exceeded that of the PED model (9.262%), while all individual-distress errors of the 4 variants (4.749%, 4.700%, 4.874%, and 4.516%) were substantially higher than the PED model’s 4.484%. The inferior performance of neural PDE and PINN variants may be partly due to their reliance on predefined or stationary governing equations, which are difficult to maintain under highly nonstationary real-world conditions (e.g., changing traffic loads and environmental stressors). In contrast, the proposed model better accommodates such nonstationarity through data-driven learning guided by mechanism-informed constraints. The residual-based PINN variant improves the predictive accuracy of the macroscopic PCI metric, but at the cost of substantially increased computational time and complexity, with a computing time of 6,423.39 s.

It is worth noting that extreme values can be observed in the area MWPE results for the PED model (45.581%). These extreme values are primarily attributed to the inherent instability of physics-informed models when applied to highly heterogeneous and nonstationary real-world deterioration data. When the model fails to accurately capture localized distress evolution, even relatively small absolute prediction errors in regions with low true values can result in disproportionately large percentage errors, thereby amplifying the area MWPE metric. Moreover, PINN-based approaches rely on predefined governing equations and boundary conditions, which are often difficult to specify consistently under complex and evolving real-world conditions. This mismatch can lead to unstable solutions in localized regions. In addition, the presence of sparse observations and measurement noise may further exacerbate sensitivity to input perturbations, resulting in large fluctuations in percentage-based error metrics.

From a practical deployment perspective, the computational overhead of the proposed PED model remains manageable. The computation time for processing about 1 year over a 160-km road network is approximately 467.17 s, demonstrating its potential for scalable urban applications, compared with 290.60 s for the neural PDE model and over 1,000 s for the 4 PINN variants. The model operates on a cell-based discretization framework, where state transitions are computed locally and aggregated across the network. This structure enables efficient scaling, as computations can be parallelized across spatial units and further benefit from ongoing improvements in computational resources. In addition, our analysis shows that a 50-d historical environmental window provides an effective balance between capturing sufficient temporal context and maintaining computational efficiency. In practical applications, this window can be extended when computational resources allow, while retaining a 50-d window remains sufficient to achieve reliable performance under constrained conditions.

These substantial computational costs of PINN variants hinder rapid iteration for city-scale DT applications. Although the neural PDE model demonstrated an advantage in computational efficiency, its prediction errors at the distress level were considerably larger. Therefore, when both predictive accuracy and computational efficiency are considered, the lightweight PED model appears more suitable for large-scale deployment and practical DT applications.

To further evaluate the robustness of the proposed model under realistic perturbations, a sensitivity comparison with state-of-the-art models was conducted. Four perturbation scenarios were considered: (a) overdetection bias (false positives), where nonexistent distress is incorrectly introduced into the model input (ratio: 0.1%); (b) underdetection bias (false negatives), where existing distress is missed in the input (ratio: 1%); (c) spatial missing, where 5% of road segments are randomly missed (masked); and (d) macro-measurement noise, where Gaussian noise with a standard deviation of 1.0 is added to the PCI observations.

The results are summarized in Table 4. Compared with the baseline results in Table 3, the PED model exhibits increased prediction errors in area MWPE under all 4 perturbation scenarios. Among them, the model demonstrates the strongest robustness to macro-measurement noise, with area MWPE increasing only slightly from 4.484% to 4.542%. In contrast, the largest performance degradation is observed under the overdetection bias scenario, where area MWPE increases to 5.540%, indicating that the PED model is relatively sensitive to falsely detected distress inputs. Under the overdetection and underdetection bias scenarios, the PCI MAPE of PED all slightly decreases, suggesting that the model may perform relatively better in macro-tasks with noise, although its overall generalization ability remains limited.

**Table 4.** Perturbation sensitivity comparison with state-of-the-art models. Bold—the best performance models. For each state-of-the-art variant, only the best-performing configuration is calculated.

State-of-the-art model	Predicted indicators/mean (min, max)							
	Overdetection bias (ratio: 0.1%)		Underdetection bias (ratio: 1%)		Spatial missing (ratio: 5%)		Macro-measurement noise (SD: 1.0)	
	Area MWPE/%	PCI MAPE/%	Area MWPE/%	PCI MAPE/%	Area MWPE/%	PCI MAPE/%	Area MWPE/%	PCI MAPE/%
**PED**	5.540(0.015, 22.889)	9.050(5.422, 14.331)	4.974(0.064, 16.145)	9.257(5.762, 14.504)	5.033(0.085, 15.933)	9.303(5.775, 14.509)	4.542(0.015, 16.624)	9.320(5.517, 14.691)
Neural PDE	67.762(2.680, 141.125)	8.269(4.990, 13.292)	45.052(0.549, 100.009)	9.044(5.616, 14.257)	39.564(0.528, 90.193)	9.131(5.609, 14.380)	45.581(0.649, 100.954)	9.074(5.423, 14.323)
fPINN (hidden dim = 128, layers = 4)	5.811(0.021, 22.889)	9.032(5.421, 14.448)	4.940(0.001, 17.235)	9.398(5.734, 14.699)	5.169(0.014, 16.642)	9.354(5.796, 14.610)	4.749(0.012, 16.245)	9.312(5.531, 14.566)
Residual-based PINN (hidden dim = 256, layers = 4)	5.757(0.018, 21.598)	9.042(5.434, 14.339)	4.695(0.030, 16.669)	9.277(5.756, 14.530)	5.445(0.033, 16.850)	9.337(5.875, 14.540)	4.700(0.026, 16.227)	9.285(5.550, 14.564)
FNN (hidden dim = 128, layers = 4)	5.609(0.005, 21.598)	9.079(5.356, 14.485)	5.003(0.052, 16.946)	9.283(5.806, 14.591)	4.531(0.103, 16.906)	9.312(5.767, 14.546)	4.874(0.079, 17.003)	9.322(5.556, 14.677)
XPINN (hidden dim = 256, layers = 4)	6.216(0.001, 22.159)	9.031(5.451, 14.371)	4.906(0.053, 16.665)	9.320(5.823, 14.639)	4.902(0.003, 17.078)	9.270(5.782, 14.637)	4.516(0.015, 17.003)	9.354(5.491, 14.739)

PCI, Pavement Condition Index; MAPE, mean absolute percentage error; area MWPE, mean weighted percentage error of area; PED, physics-embedded diffusion; PDE, partial differential equation; PINN, physics-informed neural network; fPINN, finite-difference PINN; FNN, Fourier PINN; XPINN, adaptive multidomain PINN

Although the neural PDE model consistently achieved the lowest PCI MAPE values across most disturbance scenarios, its area MWPE increased dramatically, particularly under overdetection bias. This suggests that continuous PDE diffusion tends to amplify local disturbance propagation after categorical discretization, resulting in severe instability at the microscopic distress level despite relatively accurate macroscopic PCI prediction. The extremely large maximum error values (e.g., 141.125%) further indicate that PDE-driven smoothing may excessively diffuse localized disturbance patterns into neighboring regions.

PINN-based variants exhibited relatively stable microscopic distress consistency under disturbance conditions, suggesting that physics-informed constraints help suppress excessive local error propagation. However, the improvements did not translate into superior PCI prediction accuracy.

Among all disturbance scenarios, overdetection bias produced the most severe degradation in microscopic distress consistency. This phenomenon is mainly attributed to the spatial propagation mechanism, where falsely detected distress states can diffuse to neighboring regions and accumulate during iterative evolution. On the other hand, all models show relatively low sensitivity to measurement noise, suggesting that fluctuations in macro-level statistical indicators (e.g., PCI) have a limited impact on model performance. In comparison, perturbations at the micro-level (e.g., distress state modifications) are more critical and can notably affect prediction stability.

In addition, a comparison between Fig. 4 and Fig. S3 further reveals that the PED model exhibits stable and physically consistent behavior in capturing the full life cycle of road distress, including initiation, propagation, maintenance intervention, and subsequent redevelopment. In contrast, the neural PDE variant tends to maintain macroscopic stability by underestimating the evolution of distress, which deviates from the inherently nonstationary nature of real-world spatiotemporal deterioration processes. The 4 PINN variants, as shown in Fig. S3b to e, on the other hand, tend to overestimate both the temporal progression of distress and its spatial interactions. This is primarily because such models enforce predefined physical constraints that encourage smooth and globally consistent evolution patterns, often at the expense of accurately capturing localized and heterogeneous dynamics.

### Road distress spatiotemporal evolution on the individual-distress level

To analyze the model’s applicability under specific operational scenarios, several working conditions were selected.

#### Scenario 1: Crack development and redevelopment after repair

**Fig. 6.**
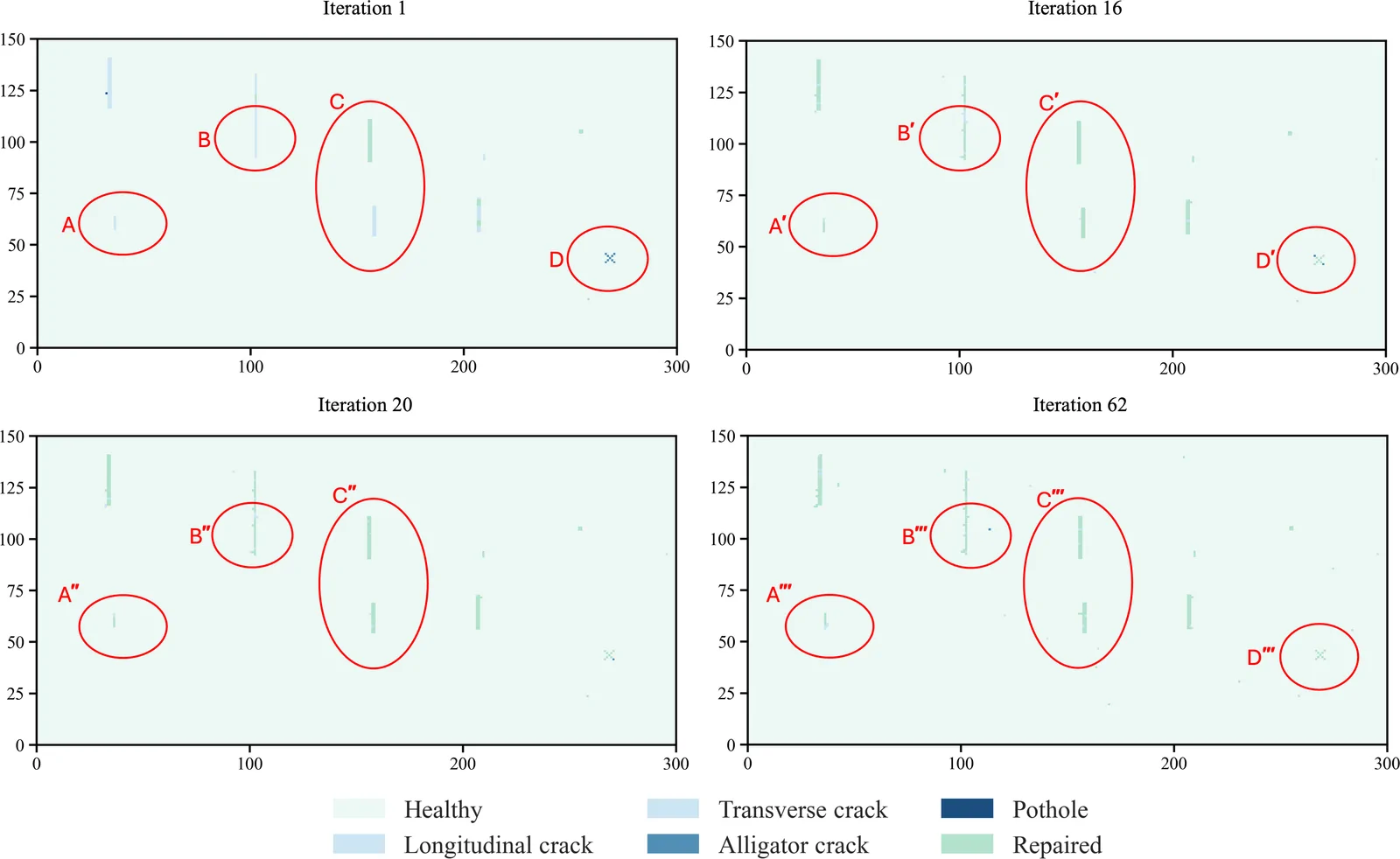
Simulation visualization of crack evolution.

Cracks are the most common form of road distress. Although crack development often progresses subtly, it can initiate the emergence of other distress types. Scenario 1 focused on the evolution of cracks. We extracted a road slice around stake −30 to 0 in the FRD-CN dataset, where longitudinal cracks, transverse cracks, and alligator cracks were the predominant distress types. The extracted slice preserved the original width dimension of 150 cells, while the longitudinal dimension was standardized to 300 cells. The visualized results are shown in Fig. 6.

As observed at point A, the transverse cracking region subsequently interacted with newly developed longitudinal cracking areas. At points B and C, the original cracks were repaired; however, potholes gradually emerged within the previously cracked regions over longer-term deterioration periods. Point D further illustrates that although the alligator cracking region underwent maintenance treatment, some minor crack regions might have been overlooked during the initial repair process. These residual defects were identified and fully repaired only in subsequent observation periods.

#### Scenario 2: Pothole development and expansion

**Fig. 7.**
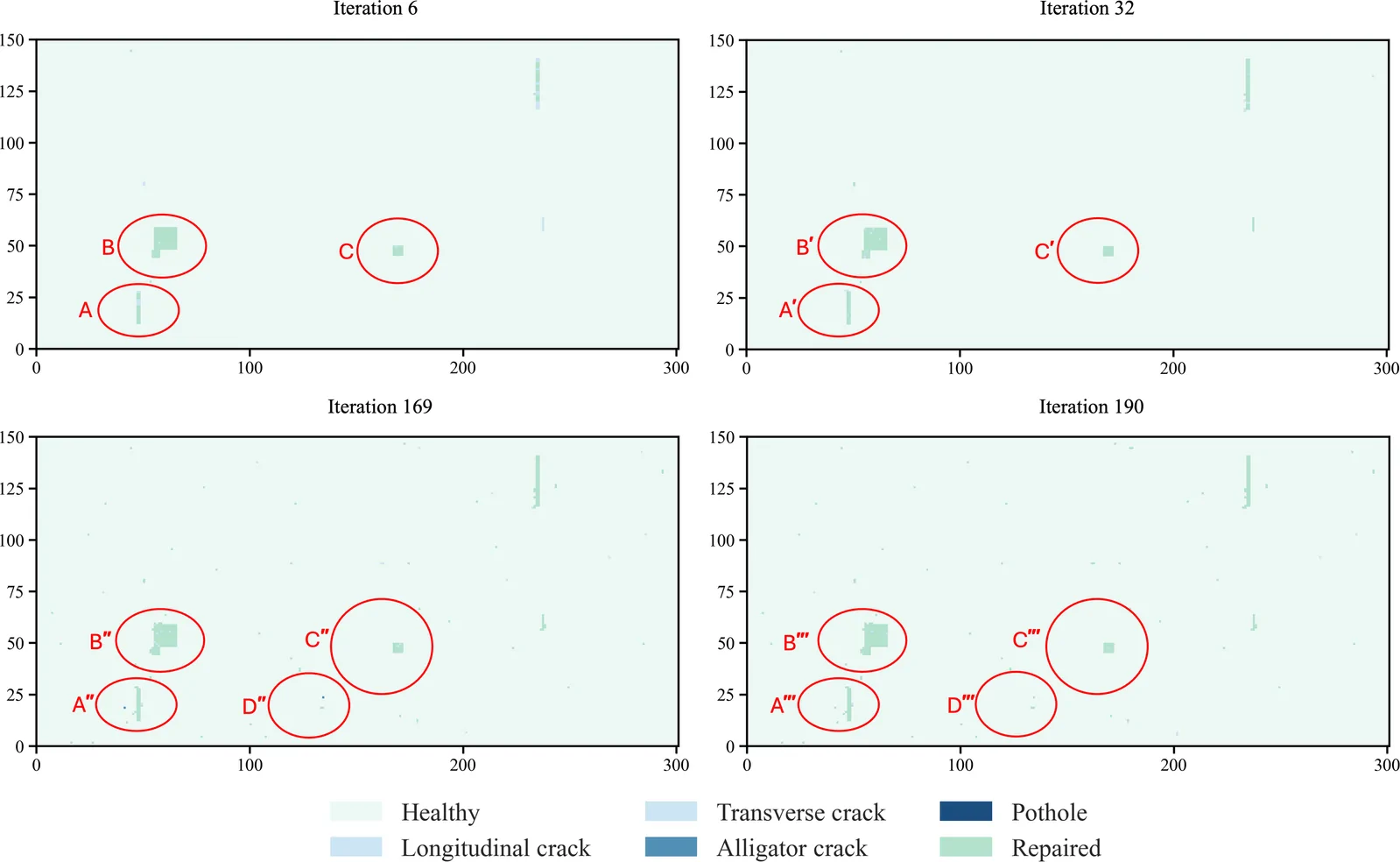
Simulation visualization of pothole evolution.

Potholes represent another critical form of road distress due to their rapid development and highly irregular progression. Timely monitoring of their spatiotemporal evolution is essential, as even localized changes in pothole conditions can substantially compromise driving safety and ride comfort. Scenario 2 focused specifically on the development and repair dynamics of potholes over time. We extracted a road slice around stakes −50 to −20, where potholes were the predominant distress types. A consistent computational domain of 150 × 300 grid cells was selected. The visualization results are presented in Fig. 7.

At point A, an initial transverse cracking region was observed. As the iterations progressed to iteration 32, the crack was repaired; however, new longitudinal cracking subsequently developed within the same area. With continued deterioration, the region eventually evolved into a pothole by iteration 169. At point B, the initial state corresponded to a previously repaired pothole region. The areas were repaired, while additional small crack regions emerged simultaneously, resulting in a repeated cycle of deterioration and maintenance. At points C and D, an increasing number of micropotholes and cracks were gradually observed over time, indicating the progressive accumulation and spatial propagation of localized road deterioration.

### Road distress spatiotemporal evolution on the section level

#### Scenario 1: Road deterioration without intervention

In the absence of maintenance, road performance follows a natural deterioration trend characterized by rapid early decline and gradual stabilization over time, consistent with the well-established patterns from the American Association of State Highway Officials Road Test, and the exponential decay model is shown in Eq. (14).

**Fig. 8.**
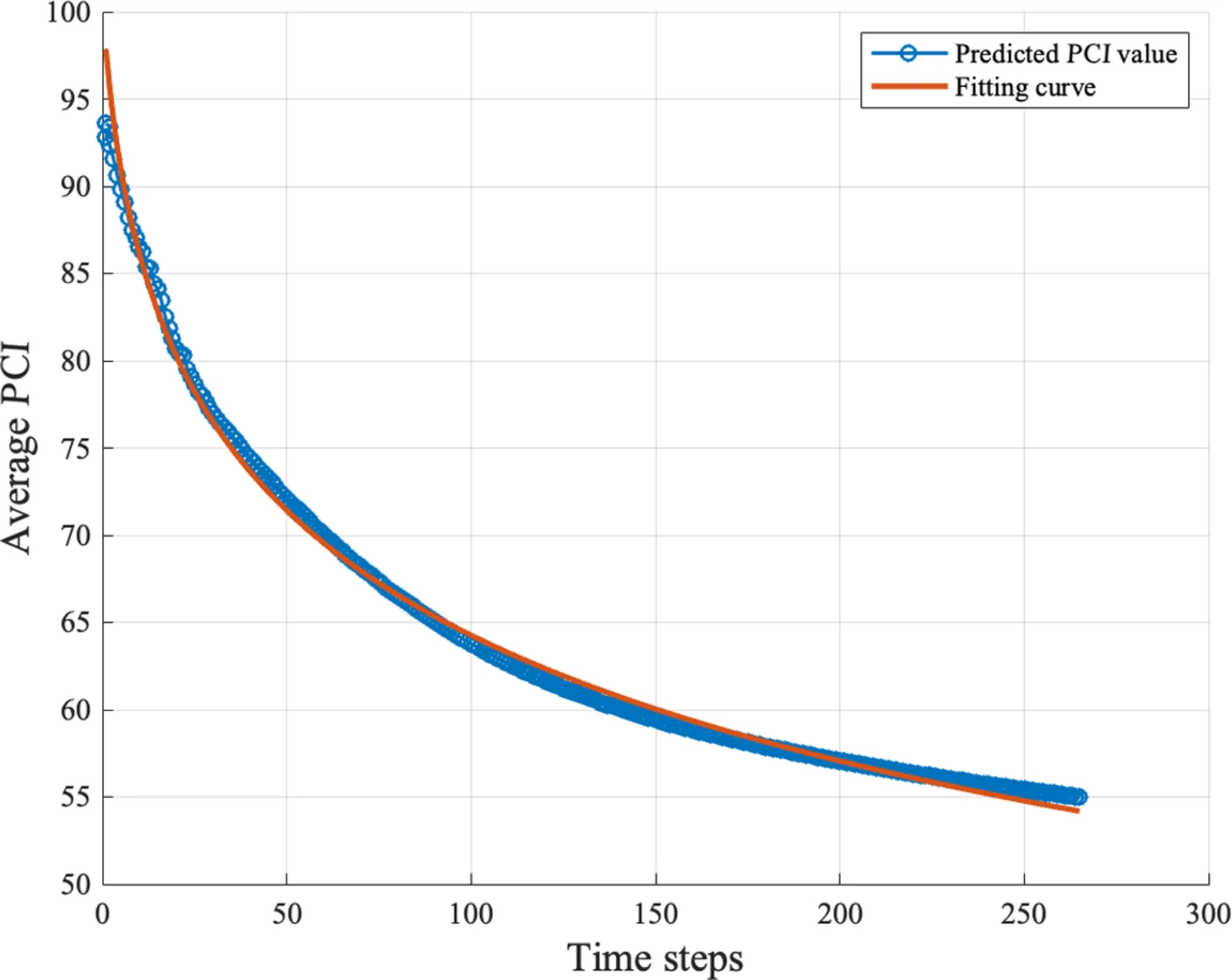
Comparison of simulation results and the standard decay equation of Pavement Condition Index (PCI).

To examine this behavior, we conducted a baseline experiment using the FRD-CN dataset without any external constraints or interventions. During training, road distress propagated continuously, and the average PCI trend across the 32 sections was computed after each iteration. As shown in Fig. 8, the simulated deterioration closely matched empirical equations. The fitted relationship (Eq. (15)) confirmed the model’s ability to reproduce realistic performance decay. However, this conclusion reflects an idealized condition without maintenance, resulting in a monotonically decreasing trend. In real-world scenarios, road performance typically exhibits oscillatory fluctuations due to periodic interventions and short-term uncertainties.

$$\mathrm{PCI}=100*\left\{1-\text{exp}\left[-{\left(\frac{\alpha }{y}\right)}^{\beta }\right]\right\}$$ (14)

$$\begin{array}{c} {\mathrm{PCI}}^{\prime }=100*\left\{1-\text{exp}\left[-{\left(\frac{110.5911}{y}\right)}^{0.2835}\right]\right\} \end{array}$$ (15)

#### Scenario 2: Road deterioration with intervention

To evaluate the model’s training performance under varying data volumes, section conditions, and maintenance histories, we conducted experiments on FRD-CN subsets comprising 1 to 32 road sections across 265 inspection timestamps.

**Fig. 9.**
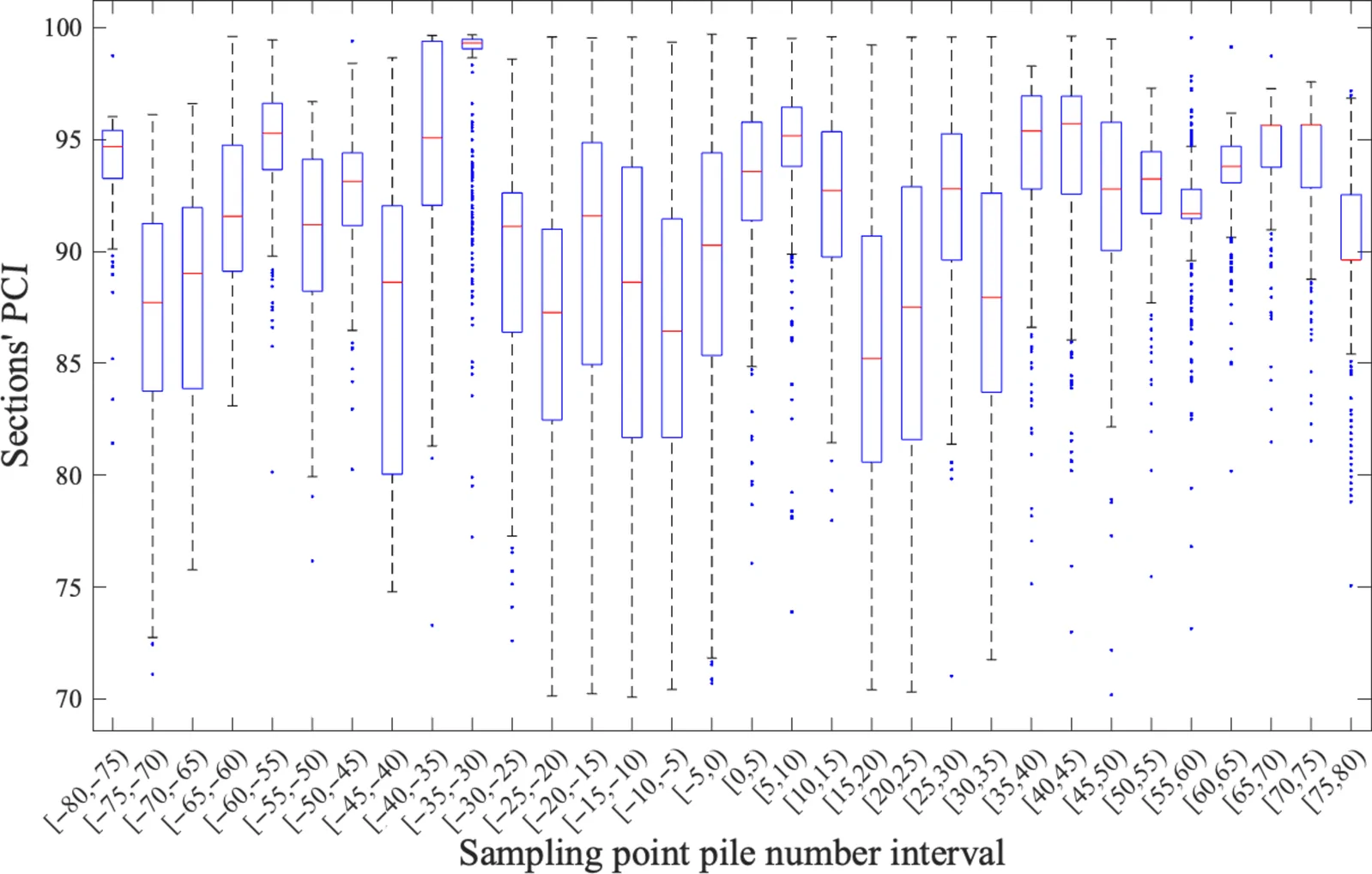
Actual performance Pavement Condition Index (PCI) distribution of multiple sections.

**Fig. 10.**
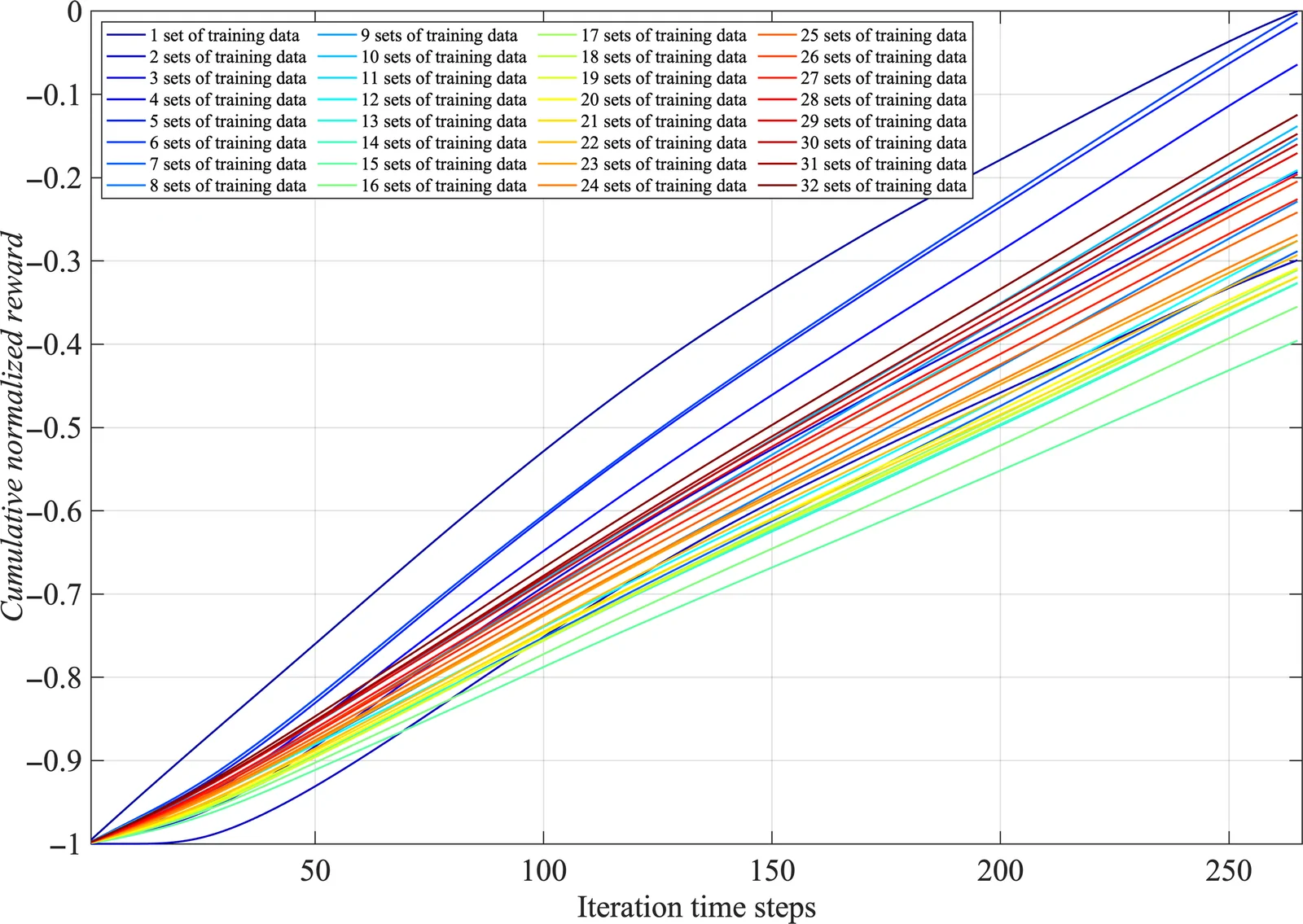
Training cumulative normalized rewards under different amounts of simulated data.

**Fig. 11.**
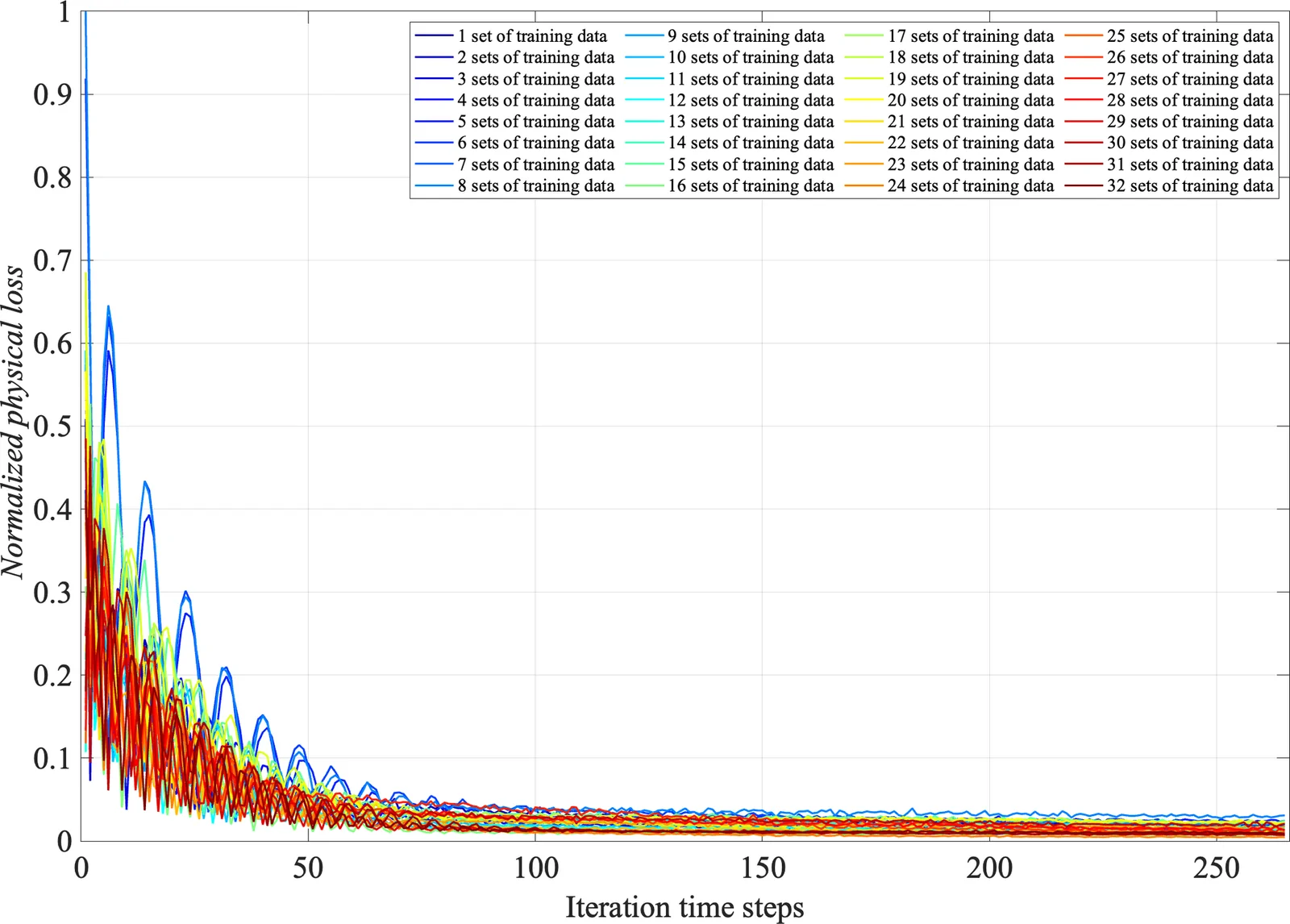
Model physical loss under different amounts of simulated data.

As illustrated in Fig. 9, the boxplot of PCI values for 32 road sections within a network shows fluctuations between 70 and 100, rather than a continuous negative exponential decay. This indicates that the PCI of typical road sections was sustained within a specific range due to major rehabilitation efforts and targeted maintenance interventions. The PCI distribution across sections was not uniform: some sections exhibited higher variability, while others showed more concentrated values, and the average PCI levels differed substantially among sections. Such heterogeneity may lead to fluctuations in model performance during training. As the dataset size increased, we compared the cumulative normalized rewards and physical loss, as illustrated in Figs. 10 and 11, respectively.

Figure 10 shows the evolution of cumulative normalized rewards over iterations, with the *x*-axis representing the number of iterations and the *y*-axis indicating the cumulative normalized reward value. All curves increase smoothly as training proceeds, indicating a consistent accumulation of rewards and a stable learning process across different amounts of training data. The trajectories remain relatively close during the early iterations but gradually separate as training progresses, reflecting differences in the rate at which individual models improve. As the number of training datasets increases, the cumulative reward does not exhibit a strictly monotonic change; it generally decreases from the lower to the intermediate data ranges before recovering as more training datasets are introduced. In particular, models trained with the largest data quantities tend to achieve higher cumulative rewards than those using intermediate-sized datasets, suggesting that the benefits of additional data become more evident after a sufficient amount of training information is available. Although some configurations achieve higher cumulative rewards more rapidly than others, the overall monotonic upward trend demonstrates that learning performance improves progressively throughout the training process.

Figure 11 presents the normalized physical loss over iterations. The training loss exhibits an overall decreasing trend with minor oscillations, primarily resulting from the exploration–exploitation trade-off. Early in training, extensive exploration led to short-term fluctuations, whereas later iterations favored exploitation and achieved faster loss reduction. With more training data, convergence accelerated, and final loss values decreased. During the early convergence phase in the 32-section simulation, the loss did not reach its absolute minimum, likely due to reduced sample efficiency as the model adapted to more diverse inputs. Nonetheless, the model ultimately achieved stable convergence, demonstrating strong learning and generalization ability under dynamic, spatially complex conditions.

### Road distress spatiotemporal evolution on the regional level

**Fig. 12.**
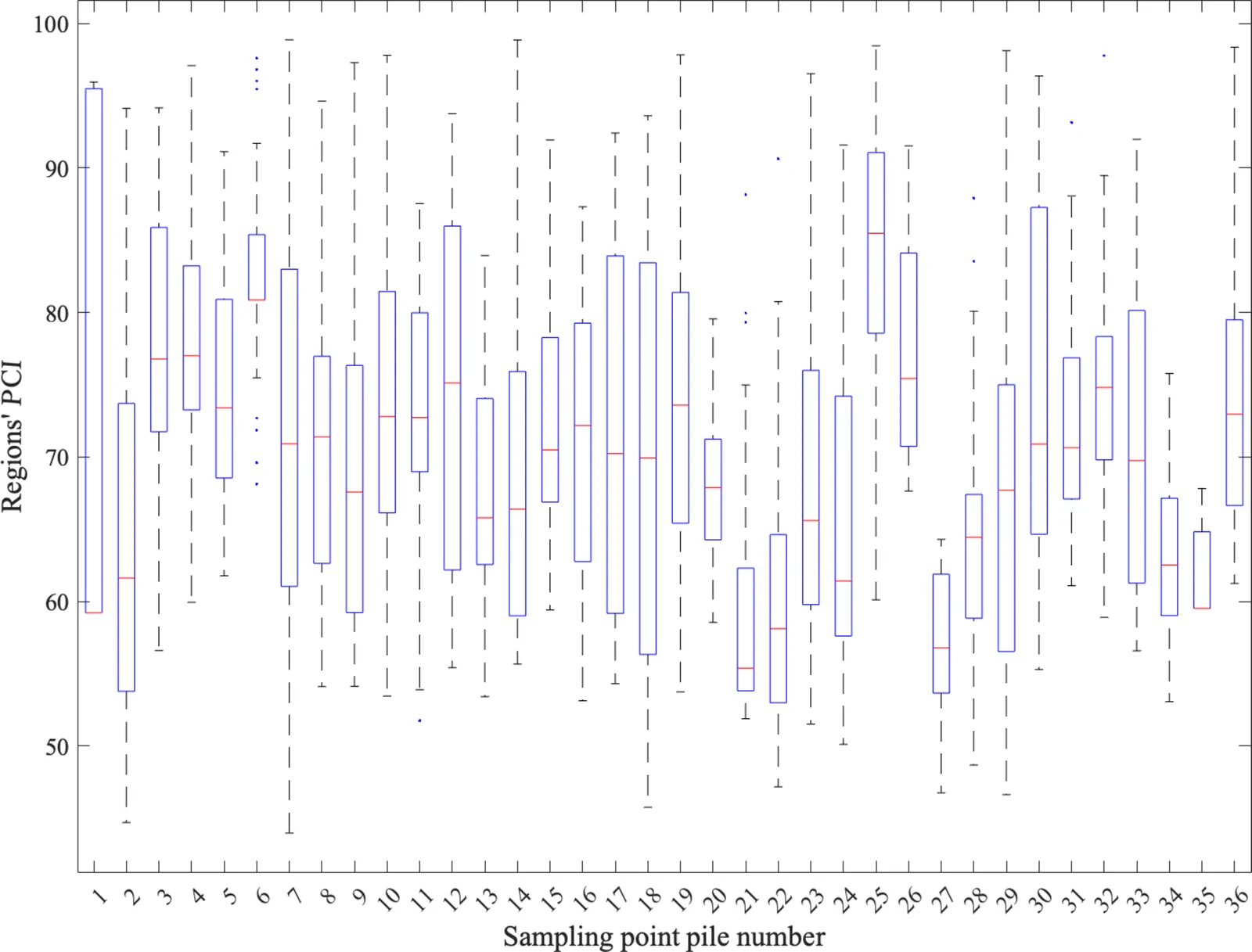
Actual performance Pavement Condition Index (PCI) distribution of multiple regions.

To evaluate the effectiveness of the proposed method over a broader spatial domain, we conducted validation on the LTPP-US dataset. The average PCI values for the 36 regions are shown in Fig. 12. Compared with the section-level fluctuations, the PCI values at the region level exhibited more pronounced oscillations and wider upper and lower bounds. This was attributable to the dataset’s longer temporal span and larger geospatial coverage, which introduced richer and more representative variations.

**Fig. 13.**
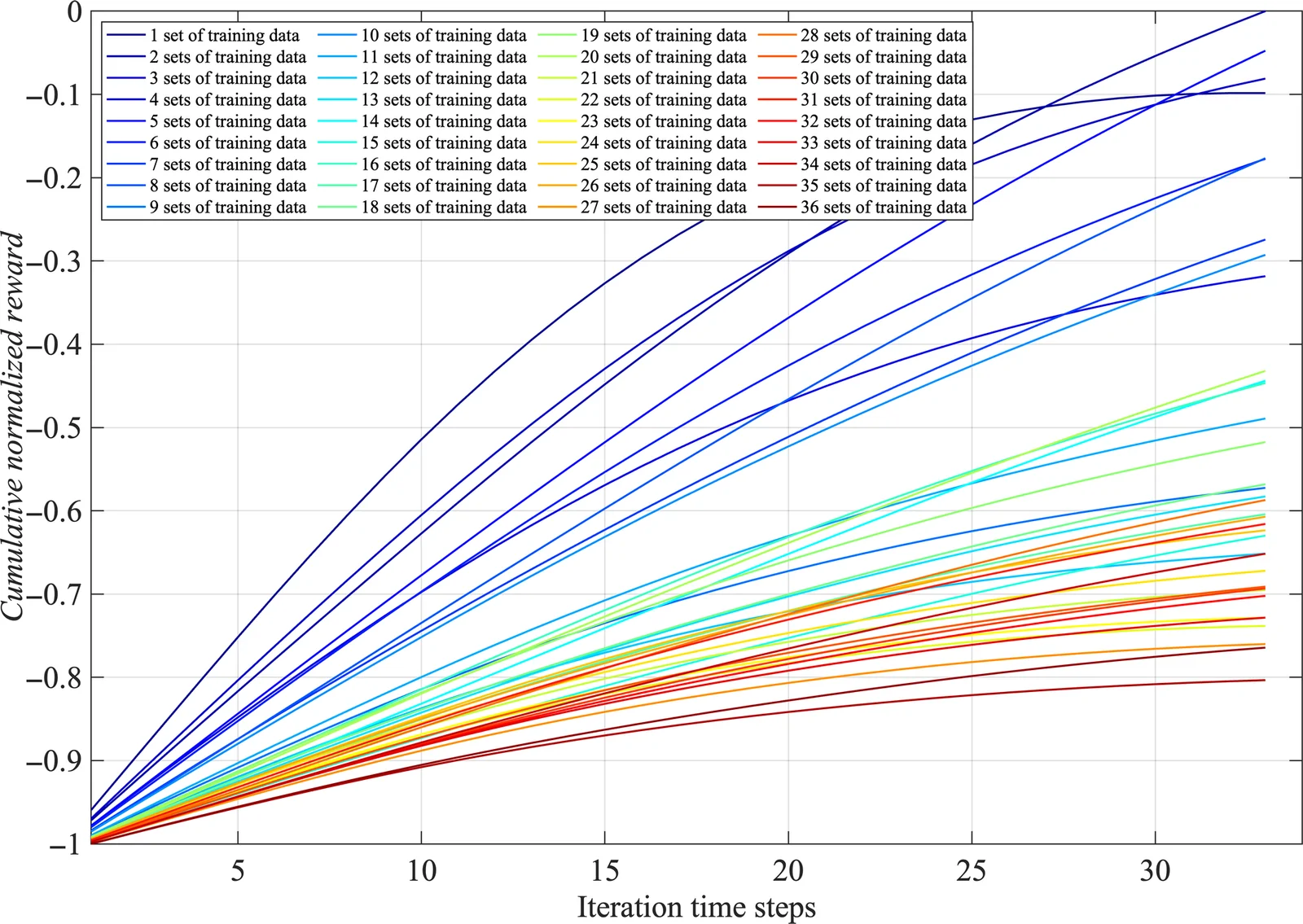
Training cumulative normalized rewards under different amounts of simulated data at the region level.

**Fig. 14.**
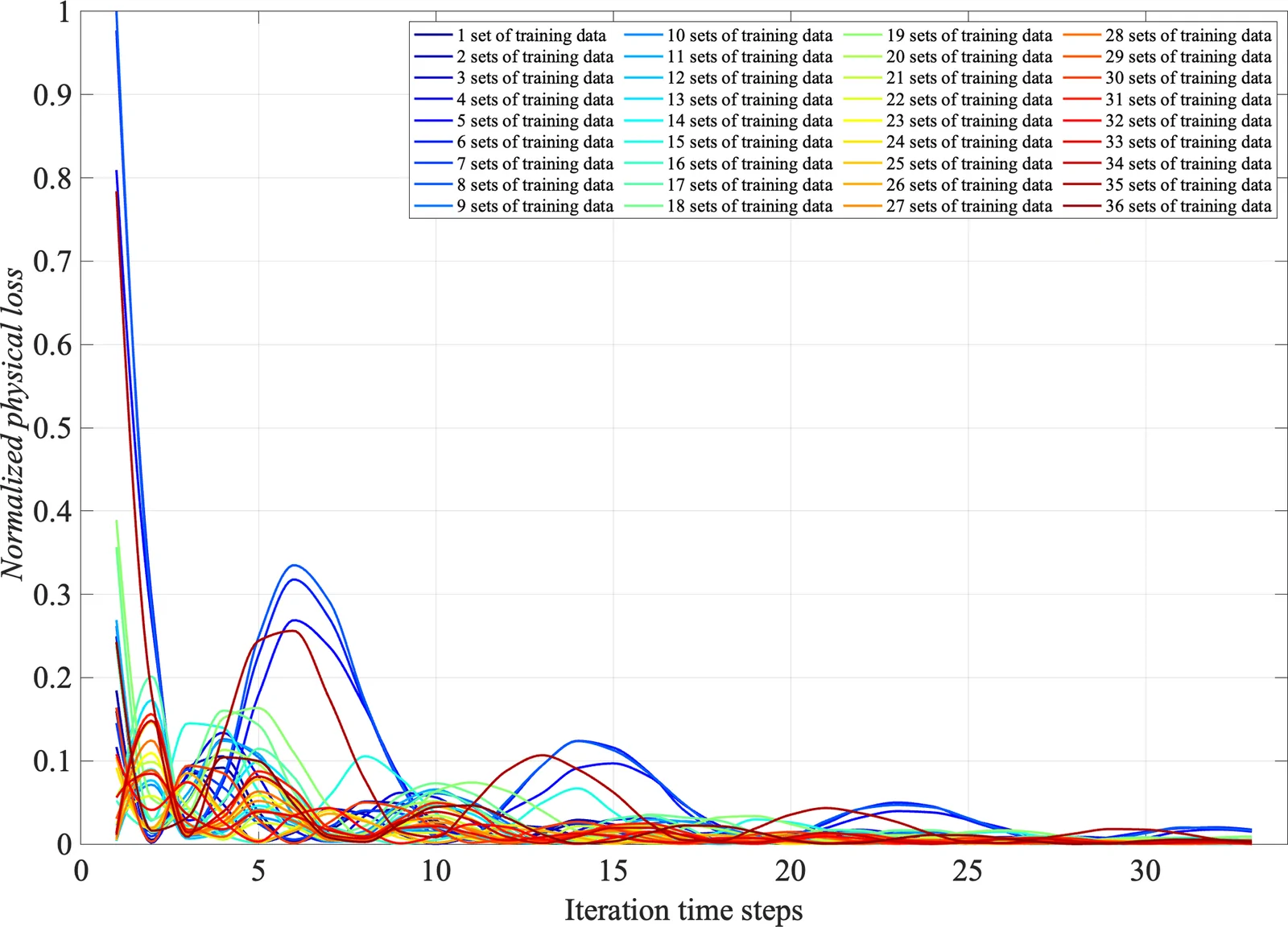
Model physical loss under different amounts of simulated data at the region level.

Figures 13 and 14 illustrate the cumulative normalized rewards and physical loss during regional-level training under different volumes of simulated data. As shown in Fig. 13, the cumulative normalized reward increases progressively with the number of iteration steps for all configurations. However, the rate of reward accumulation generally decreases as the number of simulated training regions increases. Configurations involving fewer regions exhibit steeper reward trajectories and higher final cumulative values, whereas those involving larger spatial domains tend to produce flatter curves and lower terminal rewards. This pattern suggests that increasing the number of regions introduces greater spatial heterogeneity and a more complex deterioration environment, thereby making the learning and optimization process more challenging. As shown in Fig. 14, the physical loss exhibits noticeable fluctuations during the early training stages, with several configurations showing short-lived peaks. Nevertheless, the loss decreases rapidly and converges to relatively low values as training proceeds across nearly all regional settings. These results indicate that, although expanding the simulated spatial domain increases the difficulty of reward optimization, the physics-informed training process remains numerically stable and capable of converging under different regional scales. Overall, the model demonstrates strong potential for simulating road deterioration across broader spatial domains.

## Conclusion

Accurate and fine-grained prediction of road distress is essential for informed maintenance decision-making, particularly in the context of daily operational planning. However, conventional road monitoring systems, which typically rely on data collected annually or semiannually, fail to capture the continuous and nuanced progression of road distress. Moreover, road distress evolution is a complex process influenced by both intrinsic and extrinsic factors, exhibiting individual-level uncertainty that traditional deterministic or aggregate models fail to represent. Additionally, due to intermittent interventions, the progression of road deterioration is inherently nonmonotonic, challenging the assumptions underpinning conventional regression-based approaches.

To address these limitations, this study proposes a diffusion-based DT for predicting road deterioration under climate and traffic stress to simulate the spatiotemporal evolution of road distress with improved adaptability. The proposed PED framework integrates (a) a local discrete transition module governed by neighboring states and environmental variables, (b) a continuous stochastic propagation module described by a diffusion-based generative process, and (c) physics-embedded constraints ensuring the model’s physical plausibility and extrapolation capability.

The model was trained and evaluated using the FRD-CN and LTPP-US datasets. Ablation studies demonstrated that the full model, incorporating all modules, achieved the highest predictive accuracy in the FRD-CN dataset with a PCI MAPE of 9.262% and a PCI MAPE of 28.054% in the LTPP-US dataset. At the microscopic level, the proposed model achieved an area MWPE of 4.484% on the FRD-CN dataset and 8.957% on the LTPP-US dataset, demonstrating its strong capability to accurately reproduce the spatial evolution of different pavement distress categories across both study contexts.

Compared with the state-of-the-art models, the proposed method demonstrates advantages in both microscopic modeling and computational efficiency. On the individual-distress level, the model captured key phenomena such as crack postrepair redevelopment and clustered pothole expansion, while at the section level, it successfully differentiated between deterioration patterns with and without intervention, validating the model’s applicability across scales. At the region level, it can also effectively capture the long-term deterioration mode in complex scenarios. Experiments with progressively expanded training data showed a stable convergence.

In summary, the proposed PED model demonstrates strong potential for scalable prediction of road performance dynamics. In addition, the proposed DT model builds digital entities based on physical entity information and enables performance prediction under multiple influencing factors within the digital domain. This scalable tool is invaluable for simulating realistic road deterioration processes, providing a reference for evaluating subsequent maintenance decision-making strategies. Future work will focus on further extending to simulate and evaluate network-level performance changes under different maintenance strategies, thereby supporting strategy optimization and establishing a feedback loop from digital entities back to physical assets. Future research may also build on the outputs of the proposed framework for downstream prognostic and decision-support applications. In particular, the simulated spatiotemporal deterioration trajectories generated by our model could serve as important inputs for section-level remaining useful life estimation, where dimensionality-reduction and optimized learning methods, such as kernel principal component analysis and metaheuristic-based prediction frameworks [53], may be applied. Similarly, the predicted distress-evolution patterns could provide a dynamic basis for intelligent control approaches, including U-control [54], to support maintenance planning, intervention optimization, and broader infrastructure decision-making.

## Methods

### Road distress data source

#### China Fine-grained Road Distress Dataset

**Fig. 15.**
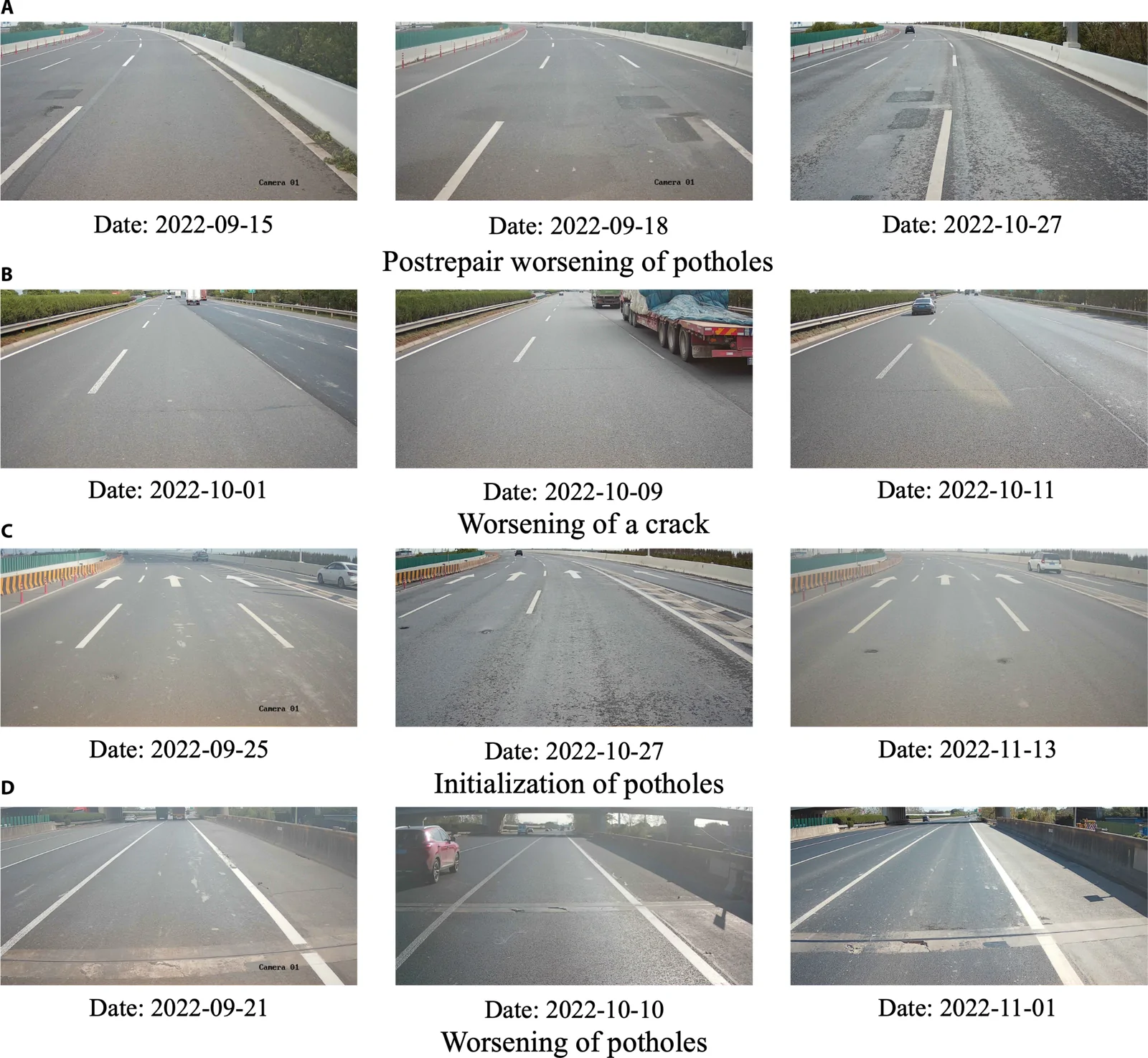
Samples of the raw spatiotemporal image data. (A) Postrepair worsening of potholes. (B) Worsening of a crack. (C) Initialization of potholes. (D) Worsening of potholes.

To collect road distress data, we integrated a vehicle-mounted system equipped with specialized devices, including high-definition industrial cameras, triaxial vibration acceleration sensors, a Global Positioning System module, and a central control unit. This system effectively captures road condition images with corresponding geospatial coordinates, timestamps, and vehicle orientation angles. The data collection was conducted periodically over 12 months, from May 2022 to April 2023, ensuring regular acquisition of asphalt road distress data along a fixed route in Shanghai, China. A total of 247 records were obtained with short sampling intervals (e.g., 1 to 2 d), comprising 60,526 road distress images (1,920 × 1,080 pixels) embedded with spatiotemporal information. Figure 15 illustrates samples of the raw spatiotemporal image data.

There were 6 types of road distress inspected, namely, cracks (both transverse and longitudinal cracks), alligator cracks, potholes, repaired cracks, repaired alligator cracks, and repaired potholes. Area information on each distress over time was extracted through manual annotation. In addition, environmental factors were also recorded, including temperature, precipitation, and traffic volume during the collection period. The meteorological data were obtained from government open platforms, while traffic volume data were collected by 462 millimeter-wave radar devices installed along the highway.

**Fig. 16.**
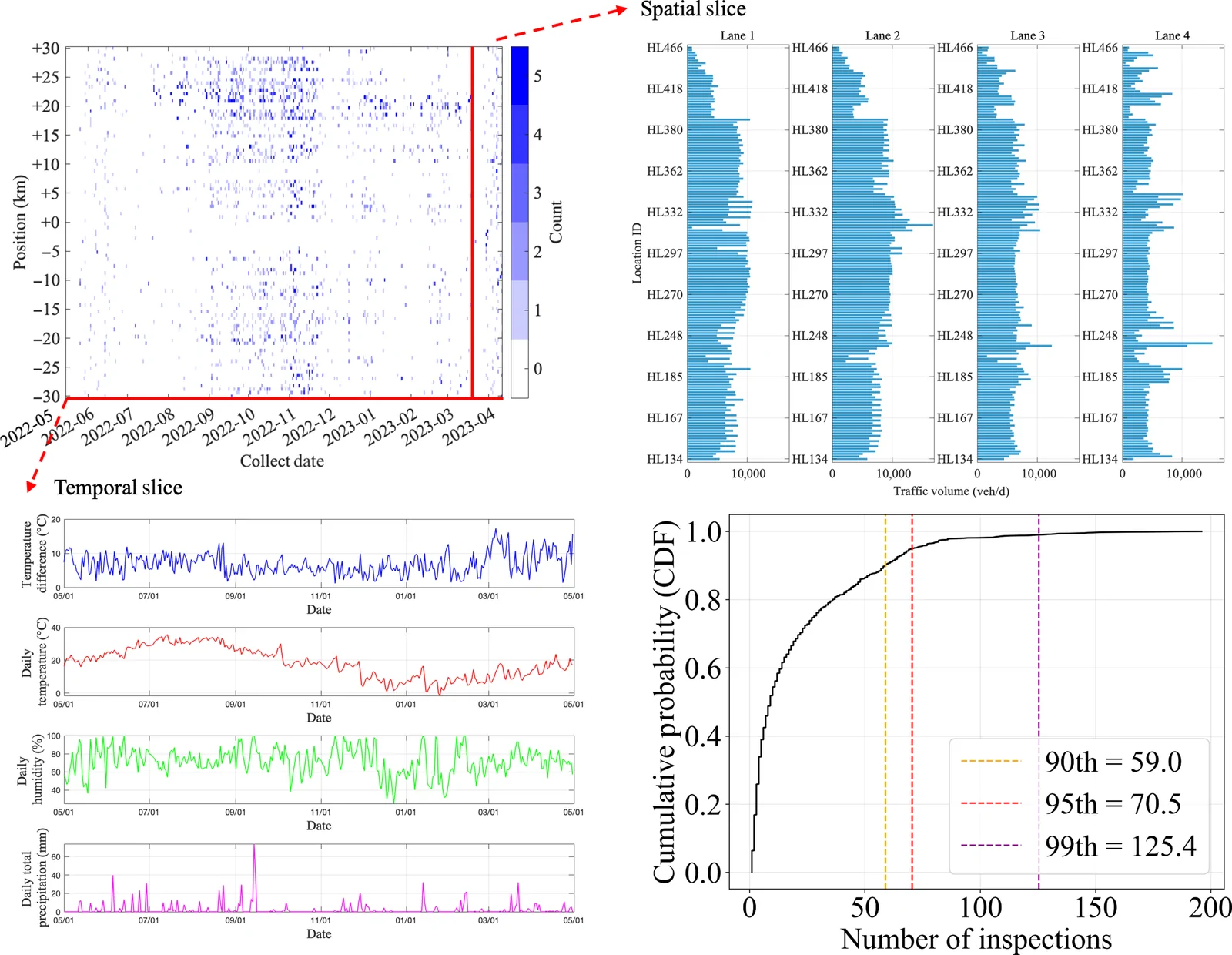
Spatiotemporal distribution of the dataset and representative samples of the environmental factors. Upper left shows the spatiotemporal distribution of the dataset with the number of repeated samples (1 to 5) at a specific spatiotemporal position. The temporal slice (bottom left) depicts the variations of temperature, temperature difference, humidity, and precipitation over time, while the spatial slice (upper right) shows the traffic volume of 4 lanes at the same collection date (the traffic volume of all lanes is assigned to the corresponding road segment). The bottom right subfigure presents the cumulative distribution function (CDF) of temporal coverage for sampling locations.

Figure 16 illustrates the spatiotemporal distribution of the dataset and representative samples of the environmental factors. The dataset contains around 800 distress points, of which we selected the top 5% with temporal coverage exceeding 70 d for model training, resulting in a reliable subset spanning 265 d of data collection. Missing values were linearly interpolated to maintain temporal continuity. After preprocessing, the input data form a 3-dimensional tensor $X\in {\mathbb{R}}^{N\times T\times F}$, where N is the number of distress sampling points, T denotes the number of historical time steps, and F represents features including areas of transverse, longitudinal, and alligator cracks; potholes; and patches and the corresponding environmental factors (temperature, precipitation, and traffic volume). Macroscopic PCI values were also computed to serve as training constraints.

#### US Long-Term Pavement Performance Dataset

The LTPP-US dataset (https://infopave.fhwa.dot.gov/), maintained by the Federal Highway Administration, provides long-term pavement performance records collected across the United States and parts of Canada. For this study, performance and environmental data from 1989 to 2023 were utilized, with temporal resolution aggregated to an annual time scale. Each record corresponds to a road section identified by its state code and geographic coordinates. The selected parameters include

• geospatial and administrative information: STATE_CODE, STATE_CODE_EXP, SURVEY_DATE, LATITUDE, and LONGITUDE
• traffic loading indicators: ANNUAL_ESAL_TREND, ANNUAL_GESAL_TREND, AADTT_ALL_TRUCKS_TREND, and ANNUAL_TRUCK_VOLUME_TREND
• environmental variables: REL_HUM_AVG_AVG, PRECIPITATION, TEMP_AVG, and TEMP_MEAN_AVG
• distress measurements: GATOR_CRACK_A, LONG_CRACK_WP_L, LONG_CRACK_NWP_L, LONG_CRACK_WP_SEAL_L, LONG_CRACK_NWP_SEAL_L, TRANS_CRACK_L, TRANS_CRACK_SEAL_L, PATCH_A, and POTHOLES_A

These variables collectively characterize traffic-induced loading, climatic conditions, and surface distress evolution for each road. Based on each section’s latitude and longitude, the study area was spatially partitioned into 36 regional zones to facilitate regional-level analysis.

### PED model architecture

**Fig. 17.**
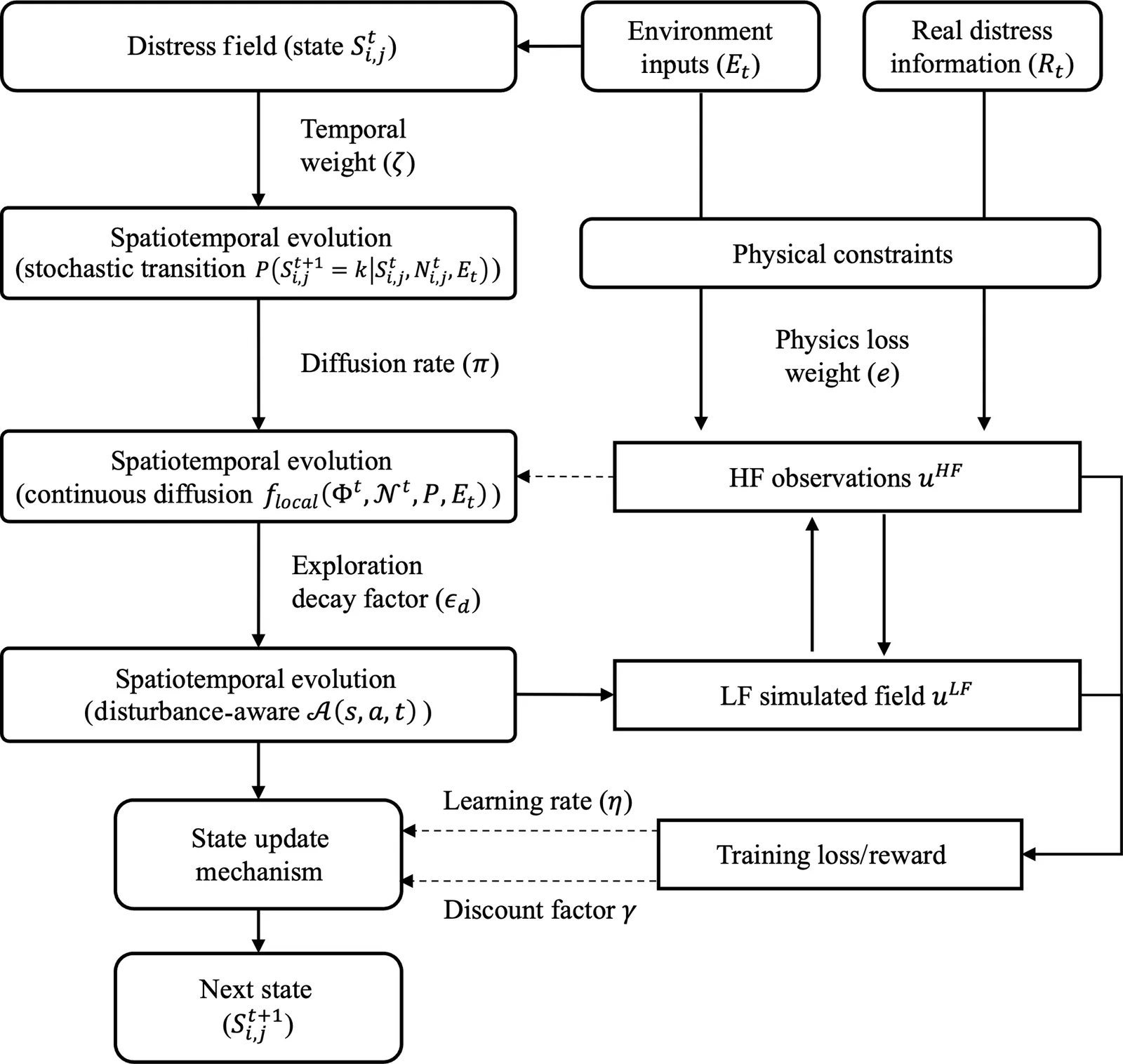
The architecture of the physics-embedded diffusion (PED) model.

The proposed PED model integrates stochastic evolution, continuous diffusion, disturbance-aware transitions, and physics-informed corrections into a unified spatiotemporal architecture for modeling road distress evolution. As illustrated in Fig. 17, the model begins with an initial distress field $S_{i,j}^{t}$, which evolves under the combined influence of environment inputs $E_{t}$ and real distress observations $R_{t}$. The evolution process is governed by 3 complementary mechanisms.

First, a discrete stochastic transition module models the short-term and neighborhood-dependent distress growth $P\left(S_{i,j}^{t+1}=k|S_{i,j}^{t},N_{i,j}^{t},E_{t}\right)$, where the temporal weight ζ regulates the contribution of historical states and environmental factors in nonstationary environments. Second, a continuous diffusion component performs localized smoothing through a physics-inspired operator $f_{\text{local}}\left(\Phi^{t},\;N^{t},\;P,E_{t}\right)$, where the diffusion rate π controls the spatial propagation strength. Third, disturbance-aware transitions $A\left(s,\;a,\;t\right)$ incorporate the impacts of maintenance actions and external events, with the exploration-decay factor $\epsilon_{d}$ determining the extent of adaptive perturbation introduced during learning-based action selection.

In parallel, physical constraints enforce consistency with governing diffusion principles using a PDE residual-based loss $L_{\text{phys}}$. The physics-loss weight ℯ balances this constraint against data-driven objectives $L_{\mathrm{obs}}$. HF observations $u^{\mathrm{HF}}$ and LF simulated fields $u^{\mathrm{LF}}$ are fused in the loss function to ensure multifidelity consistency and robust extrapolation across varying spatiotemporal regimes.

The training loss adjusts model parameters under a learning rate η. The reward is controlled by the discount factor *γ* to balance future rewards in the model’s update rule. The overall updated parameters determine the formation of the next state $S_{i,j}^{t+1}$, completing a full cycle. Together, these components allow the PED model to capture multiscale distress propagation, incorporate environmental and physical influences, and maintain stability under heterogeneous and disturbance-prone real-world conditions.

PED embeds interpretable physical priors within a data-driven framework, including local spatial dependency, diffusion-based distress propagation, deterioration–repair transitions, environmental modulation, and consistency between simulated results and observed condition indicators.

The local propagation operator $T_{\text{prop}}$ controls the stochastic spread of active distress states to neighboring cells. For a cell $c=\left(x,\;y\right)$ and one of its neighboring cells $n\in N\left(c\right)$, the transition probability can be written in a general form as

$$P\left(S_{t+1}\left(n\right)=l|S_{t}\left(c\right)=l\right)=p_{l}a_{t}E_{t}\left(y\right)G_{t}\left(y\right)\left[1+\eta_{l}D_{t}\left(n\right)\right]$$ (16)

where $l\in \left\{1,2,3,4\right\}$ represents an active distress state, $p_{l}$ is the baseline propagation probability for distress type l, $a_{t}$ is the action-dependent multiplier, $E_{t}\left(y\right)$ is the environmental scaling factor for location y, $G_{t}\left(y\right)$ is the sector-level area-consistency gate, and $D_{t}\left(n\right)$ is the diffusion spatial dependency signal. The coefficient $\eta_{l}$ controls the influence of diffusion on the corresponding distress type.

The spatial diffusion operator D represents neighborhood-dependent distress expansion. The operator generates a spatial probability field that modulates the likelihood of additional distress formation in locally susceptible regions. This can be expressed as

$$D_{t}\left(c\right)=\text{clip}\left(\frac{F_{\text{diff}}\left(S_{t},\;c\right)-1}{\delta },\;0,\;1\right)$$ (17)

where $F_{\text{diff}}\left(S_{t},\;c\right)$ is the diffusion probability factor estimated from the current cellular distress configuration, δ is a normalization constant, and $\text{clip}\left(\cdot \right)$ restricts the signal to the range $\left[0,1\right]$. This operator is used to increase the probability of distress occurrence in cells that are both spatially exposed and adjacent to existing distress clusters. Therefore, it acts as a physics-informed spatial regularization mechanism rather than a deterministic mechanical diffusion model.

The environmental scaling operator E adjusts deterioration and repair probabilities according to external exposure. Temperature, rainfall, and traffic volume are first combined into a normalized environmental exposure term $e_{t}\left(y\right)$ for each road column or sector. The scaling factor is then defined as

$$E_{t}\left(y\right)={\left[\text{clip}\left(c_{e}e_{t}\left(y\right),\;E_{\min},\;E_{\max}\right)\right]}^{\gamma }$$ (18)

where $c_{e}$ is a constant scaling coefficient; $E_{\min}$ and $E_{\max}$ define the lower and upper bounds of the environmental effect, respectively; and γ is the environmental sensitivity exponent. This operator reflects the assumption that deterioration transition probabilities should vary with environmental and loading conditions while remaining bounded to avoid unrealistic amplification.

The repair transition operator R controls transition from active distress states to the repaired state. For a cell $c=\left(x,\;y\right)$ with an active distress state $S_{t}\left(c\right)\in \left\{1,2,3,4\right\}$, the repair probability is defined as

$$\begin{array}{c} \begin{array}{c} \begin{array}{c} P\left(S_{t+1}\left(c\right)=5|S_{t}\left(c\right)\in \left\{1,2,3,4\right\}\right)=\text{clip}\left(\frac{p_{r}}{\sqrt{E_{t}\left(y\right)I_{t}\left(y\right)+\varepsilon }},\;r_{\min},\;r_{\max}\right) \end{array} \end{array} \end{array}$$ (19)

where $p_{r}$ is the baseline repair probability, $I_{t}\left(y\right)$ is the repair adjustment factor, ε is a small numerical stabilization term, and $r_{\min}$ and $r_{\max}$ define the lower and upper bounds of the repair probability, respectively. This operator represents the physical assumption that maintenance and repair activities convert active distress cells into repaired cells, while environmental exposure can influence the effective repair transition rate. Repaired cells are also allowed to reenter active distress states with a small probability, representing subsequent deterioration after treatment.

The physics residual operator introduces a regularization term for the latent deterioration field $u\left(x,\;t\right)$. The residual is defined as

$$F\left(u\right)=\frac{\partial u}{\partial t}-k\frac{\partial^{2}u}{\partial x^{2}}$$ (20)

where $u\left(x,\;t\right)$ denotes the latent deterioration intensity at location x and time t and k is a diffusion coefficient. This term encourages the learned latent deterioration field to evolve smoothly in space and time according to a diffusion-type regularity assumption. It does not imply that the model is a full continuum-mechanics road model; rather, it provides a physically motivated constraint to reduce unrealistic discontinuities in the learned deterioration process.

Finally, the equivalent-area and PCI consistency operator C links the cellular distress states to observed road condition indicators. For each sector r, the equivalent distress area of label l is calculated as

$$A_{r,t}^{\left(l\right)}=N_{r,t}^{\left(l\right)}\times \frac{\alpha_{l}^{0}+\left(\alpha_{l}^{1}-\alpha_{l}^{0}\right)\rho_{t}}{100}$$ (21)

where $N_{r,t}^{\left(l\right)}$ is the number of cells with distress label l in sector r; $\alpha_{l}^{0}$ and $\alpha_{l}^{1}$ are the start and end equivalent-area coefficients for distress type l, respectively; and $\rho_{t}=t/T$ is the normalized simulation progress. The estimated sector-level PCI is then derived from the equivalent areas using a distress-weighted transformation:

$$\begin{array}{c} {\hat{\mathrm{PCI}}}_{r,t}=100-15{\left[\frac{100\left(0.02A_{r,t}^{\left(1,2\right)}+0.01A_{r,t}^{\left(3,4\right)}+0.001A_{r,t}^{\left(5\right)}\right)}{15L_{r}}\right]}^{0.412} \end{array}$$ (22)

where $A_{r,t}^{\left(1,2\right)}$ denotes the combined equivalent area of longitudinal and transverse cracking, $A_{r,t}^{\left(3,4\right)}$ denotes the combined equivalent area of alligator cracking and potholes, $A_{r,t}^{\left(5\right)}$ denotes the repaired or patched area, and $L_{r}$ is the sector length in grid columns. This consistency operator ensures that the cell-level deterioration simulation remains linked to both observed distress-area evolution and sector-level PCI measurements.

These operators collectively define the PED module as a physics-informed deterioration representation. The stochastic transition and repair operators describe the cellular evolution of distress states; the diffusion-like spatial operator introduces neighborhood-dependent propagation; the environmental scaling operator incorporates exposure effects from temperature, rainfall, and traffic volume; the physics residual operator imposes a smooth diffusion-type regularity on the latent deterioration field; and the equivalent-area and PCI consistency operator connects cell-level simulation outputs with observed road performance indicators.

### Attention-inspired adaptive weighting of heterogeneous deterioration factors

Inspired by the principle of differentiated feature weighting in attention-based hybrid learning [55], an attention-inspired adaptive weighting mechanism was incorporated into the proposed framework to regulate the contributions of heterogeneous deterioration factors. Unlike conventional multihead self-attention, which learns query–key–value relationships from latent feature sequences, the proposed mechanism assigns physically interpretable, spatially varying weights to environmental exposure, physics-informed predictions, and local diffusion susceptibility. This design is more compatible with the cell-based deterioration process considered in this study because the model must regulate local state-transition probabilities rather than predict a single aggregated sequence-level variable.

For each longitudinal grid position y, the environmental influence factor $e_{y}$ is first adjusted according to the normalized output of the physics-informed module:

$$\begin{array}{c} {\tilde{e}}_{y}=\text{clip}\left(e_{y}\left[1+\alpha_{p}\left(\varphi_{y}-1\right)\right],\;e_{\min},\;e_{\max}\right) \end{array}$$ (23)

where $e_{y}$ denotes the combined environmental influence derived from temperature, precipitation, and traffic volume; $\varphi_{y}$ is the normalized physics-informed influence at position y; and $\alpha_{p}$ controls the contribution of the physics-informed signal. In the present implementation, $\alpha_{p}=0.2$, while $e_{\min}=0.3$ and $e_{\max}=2.0$. This formulation increases or decreases the local environmental contribution according to the spatial deterioration tendency estimated by the physics-informed component, rather than assigning an identical weight to all road cells.

The physics-informed signal is also used to adapt the local repair tendency when both the physics-informed and diffusion modules are activated:

$$a_{y}=\text{clip}\left(1+\alpha_{r}\left(\varphi_{y}-1\right),\;a_{\min},\;a_{\max}\right)$$ (24)

In this expression, $\alpha_{r}=0.2$, $a_{\min}=0.985$, and $a_{\max}=1.015$. The resulting repair probability is calculated as

$$p_{y}^{\mathrm{rep}}=\text{clip}\left(\frac{p_{0}^{\mathrm{rep}}}{\sqrt{{\tilde{e}}_{y}a_{y}+\varepsilon }},\;p_{\min}^{\mathrm{rep}},\;p_{\max}^{\mathrm{rep}}\right)$$ (25)

where $p_{0}^{\mathrm{rep}}$ is the baseline repair probability and ε is a small constant introduced for numerical stability. In the current model, the probability is bounded between 0.008 and 0.045. This formulation gives relatively greater importance to cells exposed to adverse environmental and physical conditions, for which spontaneous repair transitions should occur less frequently.

To further represent local spatial dependence, the diffusion output is transformed into a normalized susceptibility weight:

$$d_{x,y}=\text{clip}\left(\left(\frac{f_{x,y}-1}{0.03}\right)\eta_{p},\;0,\;1\right)$$ (26)

where $f_{x,y}$ is the diffusion-derived probability factor at cell $\left(x,y\right)$ and $\eta_{p}$ controls its interaction with the physics-informed component. The diffusion factor is derived from the normalized local Laplacian of the binary distress field and is restricted to a narrow interval to prevent excessive modification of the original transition process. The effective propagation probability of distress type k is subsequently expressed as

$$p_{k,x,y}^{\mathrm{eff}}=p_{k}{\tilde{e}}_{y}\left[1+g_{k}\left(t\right)d_{x,y}\right]$$ (27)

In this formulation, $p_{k}$ is the baseline propagation probability and $g_{k}\left(t\right)$ is a stage-dependent diffusion weight. A larger $d_{x,y}$ indicates stronger local spatial support for distress propagation and therefore increases the corresponding transition probability. The diffusion weights are larger during the early and intermediate stages and gradually decrease during later iterations, allowing the model to emphasize local spatial interactions during distress development while avoiding excessive diffusion after the deterioration pattern has become established.

Overall, this attention-inspired mechanism does not introduce an additional black-box attention network. Instead, it transfers the central principle of attention (differentiated weighting of information according to its relevance) to the physically interpretable transition rules of the cellular deterioration model. Environmental exposure, physics-informed estimates, and local spatial interactions are therefore weighted dynamically at each location and iteration, enabling the framework to capture heterogeneous deterioration conditions while preserving the interpretability and computational structure of the PED framework.

### Laplacian kernel

The Laplacian kernel is based on von Neumann neighborhood, using a convolution kernel to approximate the spatial Laplace operator to achieve neighborhood diffusion, as shown in Eq. (28):

$$K=\left[\begin{array}{ccc} 0.05 & 0.2 & 0.05\\ 0.2 & -1.0 & 0.2\\ 0.05 & 0.2 & 0.05 \end{array}\right]$$ (28)

The central negative weight ensures that the focal cell redistributes its value to neighbors, preserving the global mean. Larger weights for orthogonal neighbors capture primary directional diffusion, while smaller diagonal weights allow minor cross-directional smoothing. The sum-to-zero property guarantees numerical stability.

## Data Availability

The datasets generated and/or analyzed during the current study are available from the first author upon reasonable request.
