# Supplementary material for: A Diffusion-Based Digital Twin for Predicting Road Deterioration under Climate and Traffic Stress
Source: Research (Wash D C). 2026 Sep 23;9:1417. doi: 10.34133/research.1417 (PMC13598327; doi:10.34133/research.1417)
Supplement: Supplementary file 1 [file research.1417.f1.docx]

**Fig. S1.** Detailed framework of the Physics-Embedded Diffusion (PED) model for simulating the spatiotemporal evolution of road distress.

(a) PCI RMSE comparison for FRD-CN dataset

(b) PCI MAE comparison for FRD-CN dataset

(c) PCI RMSE comparison for LTPP-US dataset

(d) PCI MAE comparison for LTPP-US dataset

**Fig.** **S2.** Comparison of model performance in ablation studies across PCI RMSE, and PCI MAE.

(a) Neural PDE variant

(b) fPINN variant (hidden dim=128, layer=4)

(c) Residual-based PINN variant (hidden dim=256, layer=4)

(d) FNN variant (hidden dim=128, layers=4)

(e) XPINN variant (hidden dim=256, layer=4)

**Fig. S3.** Spatiotemporal evolution of road distress under best-performing configurations of state-of-the-art variants.

TABLE S1. Performance comparison of five variants under different parameter configurations across state-of-the-art models

| **State-of-the-art model** | **Predicted indicators / mean (min, max)** | | | | |
| --- | --- | --- | --- | --- | --- |
|  | **Area MWPE/%** | **PCI RMSE** | **PCI MAE** | **PCI MAPE/%** | **Computing Time/s** |
| PED | 4.484  (0.015, 16.213) | 10.526  (7.070, 15.487) | 8.537  (5.454, 12.974) | 9.262  (5.774, 14.460) | 467.17 |
| Neural PDE | 45.581  (0.649, 100.954) | 10.159  (6.694, 15.112) | 8.325  (5.302, 12.777) | 9.041  (5.613, 14.253) | 290.60 |
| fPINN  (hidden dim=64, layer=2) | 5.069  (0.023, 16.031) | 10.552  (7.129, 15.388) | 8.571  (5.531, 13.040) | 9.296  (5.851, 14.527) | 703.10 |
| fPINN  (hidden dim=64, layer=4) | 4.785  (0.036, 16.929) | 10.573  (7.151, 15.500) | 8.599  (5.557, 13.181) | 9.324  (5.878, 14.668) | 1065.05 |
| fPINN  (hidden dim=128, layer=2) | 5.165  (0.066, 16.508) | 10.500  (7.179, 15.444) | 8.509  (5.510, 13.054) | 9.234  (5.831, 14.542) | 734.25 |
| fPINN  (hidden dim=128, layer=4) | **4.749**  **(0.012, 16.245)** | **10.530**  **(7.137, 15.322)** | **8.551**  **(5.469, 13.009)** | **9.276**  **(5.789, 14.496)** | 1241.93 |
| fPINN  (hidden dim=256, layer=2) | 4.996  (0.001, 16.875) | 10.583  (7.091, 15.446) | 8.613  (5.462, 13.165) | 9.338  (5.783, 14.652) | 832.13 |
| fPINN  (hidden dim=256, layer=4) | 5.274  (0.004, 16.186) | 10.565  (7.169, 15.423) | 8.601  (5.509, 13.089) | 9.327  (5.830, 14.576) | 1461.45 |
| Residual-based PINN  (hidden dim=64, layer=2) | 5.063  (0.074, 16.323) | 10.556  (7.134, 15.449) | 8.570  (5.489, 13.001) | 9.295  (5.810, 14.488) | 2219.35 |
| Residual-based PINN  (hidden dim=64, layer=4) | 5.238  (0.024, 16.487) | 10.578  (7.200, 15.501) | 8.612  (5.553, 13.142) | 9.337  (5.874, 14.630) | 3263.74 |
| Residual-based PINN  (hidden dim=128, layer=2) | 5.218  (0.068, 16.929) | 10.530  (7.008, 15.400) | 8.564  (5.481, 13.026) | 9.289  (5.802, 14.514) | 3448.70 |
| Residual-based PINN  (hidden dim=128, layer=4) | 4.891  (0.072, 17.030) | 10.578  (7.088, 15.500) | 8.583  (5.446, 13.196) | 9.308  (5.766, 14.683) | 4727.93 |
| Residual-based PINN  (hidden dim=256, layer=2) | 4.880  (0.037, 16.446) | 10.548  (7.121, 15.418) | 8.541  (5.482, 12.996) | 9.266  (5.802, 14.483) | 5284.69 |
| Residual-based PINN  (hidden dim=256, layer=4) | **4.700**  **(0.026, 16.227)** | **10.516**  **(7.154, 15.479)** | **8.525**  **(5.496, 13.008)** | **9.249**  **(5.817, 14.495)** | 6423.39 |
| FNN  (hidden dim=64, layers=2) | 5.200  (0.057, 16.929) | 10.575  (7.113, 15.492) | 8.601  (5.482, 13.135) | 9.326  (5.803, 14.622) | 1113.16 |
| FNN  (hidden dim=64, layers=4) | 4.895  (0.006, 16.702) | 10.541  (7.125, 15.469) | 8.564  (5.573, 13.025) | 9.289  (5.894, 14.511) | 1292.84 |
| FNN  (hidden dim=128, layers=2) | 5.034  (0.190, 16.675) | 10.543  (7.108, 15.436) | 8.560  (5.534, 13.159) | 9.285  (5.855, 14.646) | 1177.95 |
| FNN  (hidden dim=128, layers=4) | **4.874**  **(0.079, 17.003)** | **10.555**  **(7.154, 15.414)** | **8.561**  **(5.503, 13.120)** | **9.286**  **(5.824, 14.608)** | 1428.87 |
| FNN  (hidden dim=256, layers=2) | 5.234  (0.049, 15.803) | 10.543  (7.154, 15.446) | 8.554  (5.503, 12.946) | 9.279  (5.824, 14.433) | 1356.04 |
| FNN  (hidden dim=256, layers=4) | 5.272  (0.186, 16.620) | 10.511  (7.154, 15.425) | 8.509  (5.503, 13.057) | 9.233  (5.824, 14.544) | 1750.30 |
| XPINN  (hidden dim=64, layer=2) | 5.010  (0.041, 16.797) | 10.530  (7.129, 15.384) | 8.548  (5.517, 13.095) | 9.273  (5.838, 14.583) | 1002.38 |
| XPINN  (hidden dim=64, layer=4) | 5.138  (0.005, 17.173) | 10.611  (7.155, 15.508) | 8.655  (5.577, 13.215) | 9.380  (5.898, 14.703) | 1060.77 |
| XPINN  (hidden dim=128, layer=2) | 5.013  (0.004, 16.296) | 10.555  (7.164, 15.482) | 8.578  (5.539, 13.004) | 9.303  (5.860, 14.491) | 1056.43 |
| XPINN  (hidden dim=128, layer=4) | 4.960  (0.084, 16.234) | 10.495  (7.117, 15.420) | 8.527  (5.485, 13.003) | 9.252  (5.805, 14.491) | 909.47 |
| XPINN  (hidden dim=256, layer=2) | 4.917  (0.039, 16.602) | 10.575  (7.172, 15.465) | 8.600  (5.535, 13.127) | 9.325  (5.856, 14.614) | 879.95 |
| XPINN  (hidden dim=256, layer=4) | **4.516**  **(0.015, 17.003)** | **10.567**  **(6.983, 15.515)** | **8.592**  **(5.428, 13.182)** | **9.317**  **(5.749, 14.670)** | 1172.11 |
